# Mesophyll conductance response to short‐term changes in *p* CO_2_ is related to leaf anatomy and biochemistry in diverse C_4_ grasses

**DOI:** 10.1111/nph.18427

**Published:** 2022-09-01

**Authors:** Varsha S. Pathare, Robert J. DiMario, Nuria Koteyeva, Asaph B. Cousins

**Affiliations:** ^1^ School of Biological Sciences Washington State University Pullman WA 99164‐4236 USA; ^2^ Laboratory of Anatomy and Morphology V.L. Komarov Botanical Institute of the Russian Academy of Sciences 197376 St Petersburg Russia

**Keywords:** C_4_ photosynthesis, carbonic anhydrase (CA), CO_2_ response of mesophyll conductance, leaf anatomy, mesophyll cell wall thickness, PEPC affinity for bicarbonate (*K*_m_), phosphoenolpyruvate carboxylase (PEPC), water‐use efficiency

## Abstract

- Mesophyll CO_2_ conductance (*g*_m_) in C_3_ species responds to short‐term (minutes) changes in environment potentially due to changes in leaf anatomical and biochemical properties and measurement artefacts. Compared with C_3_ species, there is less information on *g*_m_ responses to short‐term changes in environmental conditions such as partial pressure of CO_2_ (*p*CO_2_) across diverse C_4_ species and the potential determinants of these responses.
- Using 16 C_4_ grasses we investigated the response of *g*_m_ to short‐term changes in *p*CO_2_ and its relationship with leaf anatomy and biochemistry.
- In general, *g*_m_ increased as *p*CO_2_ decreased (statistically significant increase in 12 species), with percentage increases in *g*_m_ ranging from +13% to +250%. Greater increase in *g*_m_ at low *p*CO_2_ was observed in species exhibiting relatively thinner mesophyll cell walls along with greater mesophyll surface area exposed to intercellular air spaces, leaf N, photosynthetic capacity and activities of phosphoenolpyruvate carboxylase and Rubisco. Species with greater CO_2_ responses of *g*_m_ were also able to maintain their leaf water‐use efficiencies (TE_i_) under low CO_2_.
- Our study advances understanding of CO_2_ response of *g*_m_ in diverse C_4_ species, identifies the key leaf traits related to this response and has implications for improving C_4_ photosynthetic models and TE_i_ through modification of *g*_m_.

## Introduction

Mesophyll CO_2_ conductance (*g*_m_) describes the movement of CO_2_ from substomatal cavities across the intercellular air space, cell walls and membranes to the site of first carboxylation. This carboxylation occurs in the mesophyll chloroplast in species with the C_3_ photosynthetic pathway and mesophyll cytosol in species with the C_4_ photosynthetic pathway (Evans & von Caemmerer, 1996). *g*_m_ varies across plant groups due to variation in leaf anatomy and biochemistry, changes dynamically in response to environmental stimuli and has a significant impact on the plant and ecosystem‐level photosynthetic CO_2_ uptake and water‐use efficiency (Evans *et al*., 2009; Flexas *et al*., 2014; Knauer *et al*., 2019b; Pathare *et al*., 2020a). Despite its importance for photosynthetic CO_2_ uptake and water‐use at both plant and ecosystem levels and its variation across diverse plants groups, *g*_m_ is only beginning to be explicitly implemented into global models that upscale leaf‐scale photosynthetic processes to canopy and global scales (Rogers *et al*., 2017; Knauer *et al*., 2019a,b; von Caemmerer, 2021). The implementation of *g*_m_ is complicated because there is a lack of detailed information on responses of *g*_m_ to short and long‐term changes in environmental conditions (e.g. light, precipitation, temperature and CO_2_ concentration) across diverse plant groups (Rogers *et al*., 2017; Knauer *et al*., 2019a,b). Most investigations on the responses of *g*_m_ to changes in environmental conditions have focussed on C_3_ species (von Caemmerer & Evans, 2015; Xiong *et al*., 2015; Carriqui *et al*., 2018; Shrestha *et al*., 2018), but there is less information on the response of *g*_m_ to changing environmental conditions in diverse C_4_ species (Ubierna *et al*., 2017, 2018; Kolbe & Cousins, 2018; Sonawane *et al*., 2021). A better understanding of how C_4_–*g*_m_ responds to changing environmental conditions is essential for improving the models of C_4_ photosynthesis at both the leaf and global scales (Rogers *et al*., 2017; Knauer *et al*., 2019a,b; von Caemmerer, 2021) as well as for potentially increasing water‐use efficiency of C_4_ crops through manipulation of *g*_m_ (Cousins *et al*., 2020; Pathare *et al*., 2020a).

The influence of *g*_m_ on C_3_ photosynthesis has been well studied for the past several years and there has been a significant understanding of C_3_–*g*_m_ and its responses to changes in long‐term growth conditions and short‐term measurements conditions. In general, *g*_m_ varies greatly among diverse C_3_ species and limits C_3_ photosynthesis as much as stomatal conductance (*g*_sw_) (Flexas *et al*., 2014; Muir *et al*., 2014; Barbour & Kaiser, 2016; Veromann‐Jürgenson *et al*., 2017). Variation in C_3_–*g*_m_ is influenced by leaf ontogenic and anatomical traits such as leaf development and senescence (Grassi & Magnani, 2005; Barbour *et al*., 2016), surface area of chloroplasts appressed to intercellular air space (*S*_c_) (Tosens *et al*., 2012; Peguero‐Pina *et al*., 2016), mesophyll cell wall thickness (*T*_CW_) (Veromann‐Jürgenson *et al*., 2017; Ellsworth *et al*., 2018; Evans, 2021) and leaf thickness (Flexas *et al*., 2008; Muir *et al*., 2014). In terms of responses to long‐term growth conditions, C_3_–*g*_m_ is influenced by water stress, elevated CO_2_, salinity, nutrient supplement and growth latitude (Flexas *et al*., 2008; Momayyezi & Guy, 2017; Mizokami *et al*., 2018; Shrestha *et al*., 2018). Additionally, rapid responses of *g*_m_ (within minutes) have been observed in response to short‐term changes in leaf temperature, quantity and quality of light, relative humidity and CO_2_ concentrations (Hassiotou *et al*., 2009; von Caemmerer & Evans, 2015; Xiong *et al*., 2015), although not always (Loreto *et al*., 1992; Tazoe *et al*., 2009). However, there is no consensus on the exact cause of rapid responses of *g*_m_ to changes in environmental conditions such as CO_2_.

Some studies have suggested that rapid changes in *g*_m_ in C_3_ plants can be explained by changes in chloroplast position and movement that could lead to short‐term change in *S*_c_ (Oguchi *et al*., 2005; Tholen *et al*., 2008; Terashima *et al*., 2011). However, mesophyll cell wall thickness (*T*_CW_) and composition are considered invariable in the short term (minutes) and may not explain the rapid responses of *g*_m_ (Evans *et al*., 2009; Terashima *et al*., 2011; Carriqui *et al*., 2018). Alternatively, the rapid responses of C_3_–*g*_m_ have also been attributed to changes in activities of key photosynthetic enzymes such as carbonic anhydrase (CA), which catalyses the conversion of CO_2_ to HCO_3_^−^ (Evans *et al*., 2009; Momayyezi & Guy, 2017) and the facilitation effect of CO_2_‐permeable aquaporins (Uehlein *et al*., 2008; Flexas *et al*., 2012; Kaldenhoff, 2012; Groszmann *et al*., 2017; but please refer to Kromdijk *et al*., 2019; Huang *et al*., 2021). Still, other groups have suggested that the rapid responses of C_3_–*g*_m_ could be the result of systematic methodological errors or the use of oversimplified models. These include mathematical dependency of *g*_m_ on other variables (such as *A*_net_ and *C*_i_) used to calculate it in fluorescence and Δ^13^C methods, neglecting the contribution of respiratory and photorespiratory CO_2_ release to the total CO_2_ pool in the leaf and inaccurate measurement of day respiration or estimates of the Rubisco fractionation factor in the Δ^13^C method (Tholen & Zhu, 2011; Gu & Sun, 2014; Carriqui *et al*., 2018; Ubierna *et al*., 2019). The exact mechanism underlying the CO_2_ responses of C_3_–*g*_m_ therefore remains controversial. However, considerable evidence based on diverse species and methods of estimating *g*_m_ have suggested that the C_3_–*g*_m_ values increase at low partial pressures of CO_2_ (*p*CO_2_).

There has been a recent increase in research on the short‐term and long‐term variability of *g*_m_ in diverse C_4_ species and in the leaf traits that could explain this variability (Barbour *et al*., 2016; Cano *et al*., 2019; Pathare *et al*., 2020a,b). Our recent study demonstrated that, as for C_3_ species, *g*_m_ varied significantly among diverse C_4_ grasses and had significant effects on photosynthetic rates and leaf water‐use efficiencies (Pathare *et al*., 2020a). This variation in C_4_–*g*_m_ was correlated with leaf‐level traits such as leaf thickness, stomatal ratio (SR), adaxial stomatal densities and S_mes_. We also demonstrated that C_4_ grasses adapted to low precipitation habitats exhibited traits related to greater *g*_m_ but lower leaf hydraulic conductance compared with grasses from habitats with relatively high precipitation (Pathare *et al*., 2020b). These studies have advanced our understanding of the variability of *g*_m_ in C_4_ grasses, as well as how *g*_m_ is influenced by leaf‐level traits and is affected by long‐term growth conditions such as precipitation. However, there is still a limited understanding of how *g*_m_ in diverse C_4_ grasses responds to short‐term changes in environmental conditions such as CO_2_. The few previous studies have largely focussed on a few species such as sorghum, maize and *Setaria* (Osborn *et al*., 2017; Kolbe & Cousins, 2018; Ubierna *et al*., 2018; Sonawane & Cousins, 2020). To the best of our knowledge, no studies to date have explored if *g*_m_ responses to short‐term changes in *p*CO_2_ vary among diverse C_4_ grasses and what potential anatomical and biochemical traits could explain this variation in CO_2_ response of C_4_–*g*_m_.

The overall objectives of the current study were (1) to investigate the response of *g*_m_ to short‐term changes in *p*CO_2_ in diverse C_4_ species, (2) identify the anatomical and biochemical traits that may explain the variable CO_2_ response of C_4_–*g*_m_, and (3) evaluate the impact of varying CO_2_ responses of *g*_m_ on changes in photosynthesis and leaf water‐use efficiency. To address the above objectives, we estimated *g*_m_ under changing *p*CO_2_ in 16 diverse C_4_ grasses (please refer to Pathare *et al*., 2020a for details), using the most recent method described by Ogee *et al*. (2018). We also investigated the relationship of leaf anatomical traits, previously known to influence C_4_–*g*_m_, with variable CO_2_ responses of *g*_m_ in the 16 C_4_ grasses. Furthermore, we investigated the impacts of photosynthetic capacity on the CO_2_ response of *g*_m_ in these C_4_ grasses through measurements of maximum photosynthetic rates (*A*_max_), leaf nitrogen content (N_area_), activities of key enzymes of C_4_ photosynthetic pathway such as CA, phosphoenolpyruvate carboxylase (PEPC) and ribulose‐1,5‐bisphosphate carboxylase/oxygenase (Rubisco) and PEPC affinity for its substrate HCO_3_^−^ (*K*_m_). N_area_ is integral to the proteins of photosynthetic machinery (such as PEPC, Rubisco and CA) that, along with the leaf structure, are responsible for the drawdown of CO_2_ inside the leaf (Parkhurst, 1994; Wright *et al*., 2004; Evans *et al*., 2009; DiMario *et al*., 2018).

## Materials and Methods

### Plant growth

Sixteen C_4_ grasses (Table 1) were selected for this study. In the graphs, each species is identified by four letter word that combines the first letter of the genus and first three letters of the species name. The plants were raised from seeds and grown in 3‐l free drainage pots in a controlled environment growth chamber (model GC‐16; Enconair Ecological Chambers Inc., Winnipeg, MB, Canada). The photoperiod was 14 h including a 2 h ramp at the beginning and end of the light period. Light and dark temperatures were maintained at 26 and 22°C, respectively. Light was provided by 400‐W metal halide and high‐pressure sodium lamps with maximum photosynthetic photon flux density (PPFD) of *c*. 1000 μmol photons m^−2^ s^−1^ at plant height. One individual per species was grown per pot in a Sunshine mix LC‐1 soil (Sun Gro Horticulture, Agawam, MA, USA) with five or six replicate pots per species. The plants were irrigated daily to pot saturation and fertilised twice a week with Peters 20–20–20 (2.5 g l^−1^). Pots were randomised daily within the growth chamber.

**Table 1:** Mean ± SE (*n* = 3–6) values along with the corresponding letters of *post‐hoc* Tukey's test for important leaf‐level anatomical and biochemical traits measured for the 16 C_4_ grasses.

Species	Species code	*T*_CW_ (μm)	S_mes_ (μm^2^ μm^−2^)	SR (unitless)	SD_ada_ (number mm^−2^)	Leaf thickness (μm)	PEPC activity (μmol m^−2^ s^−1^)	Rubisco activity (μmol m^−2^ s^−1^)	*k*_CA_ (μmol m^−2^ s^−1^ Pa^−1^)	*K*_m_ (μM HCO_3_^−1^)	*A*_max_ (μmol CO_2_ m^−2^ s^−1^)	N_area_ (gm^−2^)
*Chloris gayana*	cgay	0.09 ± 0.012 a	9.3 ± 0.5 bcf	0.62 ± 0.08 c	106.2 ± 6.74 fhi	153 ± 9.4 bc	112 ± 13 bf	32 ± 3 bh	19 ± 1 cdeg	29 ± 0.8 ad	35.6 ± 1 abc	1.81 ± 0.16 abcd
*Danthoniopsis dinteri*	ddin	0.11 ± 0.005 ab	18.6 ± 1.3 a	1.45 ± 0.08 ab	142.9 ± 4.5 abc	241 ± 1.7 a	156 ± 8 bc	81 ± 4 a	49 ± 4 ab	33 ± 1.4 abcd	42.4 ± 1.1 a	2.25 ± 0.07 a
*Digitaria sanguinalis*	dsan	0.11 ± 0.009 abcd	8.9 ± 0.6 bc	0.52 ± 0.02 c	40.4 ± 3.26 de	155 ± 8.3 bc	721 ± 33 a	30 ± 1 b	13 ± 2 cde	44 ± 1.6 ef	33.2 ± 2.8 bcde	1.5 ± 0.09 bcd
*Eriachne aristidea*	eari	0.08 ± 0.004 a	13.1 ± 0.2 de	2.33 ± 0.11 d	130 ± 5.53 abf	216 ± 3.6 ad	348 ± 46 de	79 ± 4 ac	74 ± 6 af	36 ± 1.1 abceg	37.7 ± 2.2 abc	2.22 ± 0.09 a
*Echinochloa colona*	ecol	0.11 ± 0.006 abc	10 ± 0.3 bcdef	0.82 ± 0.02 ce	68.7 ± 4.17 dg	168 ± 3.5 bef	408 ± 11 de	50 ± 2 de	72 ± 1 af	46 ± 2.1 f	42.5 ± 0.9 a	1.71 ± 0.03 abcd
*Eragrostis curvula*	ecur	0.17 ± 0.03 cdef	10.6 ± 0.6 bcdef	1.09 ± 0.07 ef	97.8 ± 7.39 hi	176 ± 17.9 bdef	97 ± 14 f	43 ± 0 dfgh	35 ± 2 bg	34 ± 0.7 abcdg	38 ± 0.7 abc	2.22 ± 0.22 a
*Eleusine indica*	eind	0.12 ± 0.006 abcde	10 ± 0.4 bcdef	1.41 ± 0.07 abf	155.8 ± 2.91 ac	186 ± 1.8 bdef	149 ± 15 bcf	41 ± 3 bdfgh	14 ± 2 cde	33 ± 1.3 abcd	41.1 ± 0.5 ab	2.05 ± 0.14 abc
*Eriochloa sericea*	eser	0.1 ± 0.001 abc	11.3 ± 0.4 bcdef	1.41 ± 0.11 af	89.8 ± 2.55 gh	184 ± 2.8 bdef	496 ± 16 ad	59 ± 2 ce	86 ± 2 f	41 ± 0.9 efg	35.5 ± 2.8 abc	2.08 ± 0.1 ac
*Heteropogon contortus*	hcon	0.18 ± 0.004 ef	9.9 ± 0.3 bcdf	0.56 ± 0.02 c	53.9 ± 2.98 d	119 ± 8.5 c	116 ± 21 bf	36 ± 2 bfgh	9 ± 1 c	35 ± 1.2 abcdg	25.6 ± 2.7 de	1.24 ± 0.07 d
*Ischaemum afrum*	iafr	0.1 ± 0.012 a	12.4 ± 0.3 def	0.81 ± 0.03 ce	157.3 ± 10.11 c	153 ± 5.3 bc	323 ± 28 de	48 ± 2 deg	25 ± 1 deg	39 ± 0.9 cefg	33.6 ± 1.6 bcd	1.43 ± 0.08 d
*Paspalum dilatatum*	pdil	0.21 ± 0.005 f	8.5 ± 0.3 b	0.68 ± 0.05 c	52 ± 3.62 d	150 ± 9.7 bc	185 ± 8 cg	38 ± 1 bdfgh	15 ± 3 cde	31 ± 0.9 abd	30.6 ± 0.4 cde	1.39 ± 0.1 d
*Paspalum macrophyllum*	pmac	0.18 ± 0.022 def	9.8 ± 0.4 bcdf	0.12 ± 0.01 g	12 ± 1.04 e	159 ± 6.2 bce	162 ± 8 bc	30 ± 2 b	12 ± 1 cd	37 ± 2.2 bceg	25.3 ± 1.1 e	1.47 ± 0.05 bd
*Panicum maximum*	pmax	0.12 ± 0.005 abcd	11.9 ± 0.2 cdef	1.76 ± 0.12 b	111.8 ± 5.14 fhi	161 ± 2.4 bce	155 ± 9 bcf	44 ± 3 dfg	69 ± 5 af	30 ± 2.7 abd	38.1 ± 0.9 abc	1.72 ± 0.01 abcd
*Panicum miliaceum*	pmil	0.11 ± 0.001 abc	11.8 ± 1.4 cdef	1.31 ± 0.04 af	88.6 ± 4.52 gh	212 ± 11.4 ad	199 ± 17 cg	31 ± 2 b	78 ± 3 f	30 ± 1.1 abd	38.6 ± 0.4 abc	1.77 ± 0.04 abcd
*Panicum virgatum*	pvir	0.17 ± 0.03 bcdef	13.6 ± 1.3 e	1.28 ± 0.04 af	117.3 ± 7.89 bfi	210 ± 12.9 adf	296 ± 38 eg	62 ± 5 ace	165 ± 22 h	29 ± 0.2 d	36.9 ± 1.5 abc	2.27 ± 0.22 a
*Urochloa dictyoneura*	udic	0.08 ± 0.003 a	9.9 ± 0.6 bcdf	1.11 ± 0.03 ef	129 ± 5.08 abf	201 ± 4.6 adef	154 ± 8 bcf	34 ± 2 bfh	29 ± 2 beg	46 ± 1.7 f	37.4 ± 0.6 abc	1.29 ± 0.05 d

Species code was created using first letter of the genus and first three letters of the species. Results of one‐way ANOVA are given in Supporting Information Table S2. Information on species biochemical subtypes can be found in Table S3. Values for *T*_CW_, S_mes_, SR, SD_ada_, Leaf thickness, *k*_CA_ and N_area_ have been published in Pathare *et al*. (2020a), whereas values for *K*_m_ have been published in DiMario *et al*. (2021). Mesophyll cell wall thickness (*T*_CW_), total mesophyll cell surface area exposed to intercellular air space per unit of leaf surface area (S_mes_), stomatal ratio (SR), adaxial stomatal density (SD_ada_), phosphoenolpyruvate carboxylase activity (PEPC), carbonic anhydrase activity expressed as first‐order rate constant (*k*_CA_), PEPC affinity for HCO^−^_3_ (*K*_m_), maximum photosynthetic capacity (*A*_max_) and leaf N content (N_area_).

### Gas exchange measurements and estimation of *g*_m_

The measurements of net photosynthetic rates (*A*_net_, μmol CO_2_ m^−2^ s^−1^), stomatal conductance to water vapour (*g*_sw_, mol water m^−2^ s^−1^), intercellular CO_2_ concentrations (*C*_i_, Pa) and mesophyll conductance to CO_2_ (*g*_m_, μmol m^−2^ s^−1^ Pa^−1^) were performed at four different CO_2_ levels inside the chamber or *C*_a_ (34, 27, 20 and 14 Pa) using an LI‐6400XT infrared gas analyser (Li‐Cor, Lincoln, NE, USA). Intrinsic water‐use efficiency (TE_i_, μmol CO_2_ mol^−1^ water) was calculated as *A*_net_/*g*_sw_ for each CO_2_ level. Maximum photosynthetic rates (*A*_max_, μmol CO_2_ m^−2^ s^−1^) were measured at saturating light of *c*. 1200 μmol photons m^−2^ s^−1^ and *p*CO_2_ of *c*. 1500 μmol mol^−1^.

Several methods have been used to estimate C_4_–*g*_m_. Pfeffer & Peisker (1998) calculated the *g*_m_ from the initial slope of a photosynthetic CO_2_ response curve and assumed no CO_2_ dependence of *g*_m_. However, C_4_–*g*_m_ is sensitive to *p*CO_2_ (Kolbe & Cousins, 2018; Ubierna *et al*., 2018) and therefore the initial slope method may be problematic. Using anatomical traits such as S_mes_ for estimating C_4_–*g*_m_ could also be subject to errors due to assumptions made for values of *T*_CW_, porosity and membrane permeability (Pengelly *et al*., 2010). The ∆^13^C *in vitro V*_pmax_ method (Ubierna *et al*., 2017) estimates C_4_–*g*_m_ by retrofitting models of C_4_ photosynthesis model (von Caemmerer, 2000) and the ∆^13^C (Farquhar & Cernusak, 2012) with gas exchange, kinetic constants and *in vitro* PEPC activities. The ∆^13^C *in vitro V*_pmax_ method may also result in inaccurate estimates of C_4_–*g*_m_ due to errors associated with the estimation of *in vitro* PEPC and CA activities and enzyme kinetic parameters. The ∆^18^O method (Gillon & Yakir, 2000; Barbour *et al*., 2016; Barbour & Kaiser, 2016) utilises simultaneous measurements of the oxygen isotope composition (δ^18^O) of transpired H_2_O and oxygen isotope discrimination (Δ^18^O) in CO_2_ to calculate the CO_2_ concentration at the site of isotope exchange by CA. The Δ^18^O method assumes full isotopic equilibrium between CO_2_ and H_2_O at the site of CA (*θ* = 1), which may not always be true and therefore *g*_m_ could be underestimated. In the current study we used the method described by Ogee *et al*. (2018) that builds upon the Δ^18^O method (Barbour *et al*., 2016; Ogee *et al*., 2018). This method utilises a newly developed model of C_4_ photosynthetic discrimination that provides an estimate of the isotopic equilibrium between CO_2_ and H_2_O inside the leaf and *g*_m_ and accounts for the physical separation between mesophyll and bundle sheath cells in C_4_ leaves and the contribution of respiratory fluxes (Ogee *et al*., 2018). For estimating *g*_m_, isotopologs of CO_2_ and H_2_O were measured using a LI‐6400XT infrared gas analyser (Li‐Cor) coupled to a tunable diode laser absorption spectroscope (TDLAS, model TGA 200A; Campbell Scientific, Logan, UT, USA) and a cavity‐ring down absorption spectroscope (Picarro, Sunnyvale, CA, USA), as described previously (Ubierna *et al*., 2017; Kolbe & Cousins, 2018; Pathare *et al*., 2020a). The entire LI6400XT, the 2 cm × 6 cm leaf chamber (6400‐11; Li‐Cor) and LI‐6400‐18‐RGB light source were placed in a growth cabinet (model EF7, Conviron; Controlled Environments Inc., North Branch, MN, USA) with fluorescence lamps (F48T12/CW/VHO; Sylvania, Wilmington, MA, USA) set at a PPFD of *c*. 250 μmol photons m^−2^ s^−1^ and air temperature was maintained at 25°C. For each species, four‐point CO_2_ response curve of *g*_m_, *g*_sw_, *A*_net_ and TE_i_ was performed. During measurements CO_2_ sample (CO_2_S) was set to *c*. 34, 27, 20 and 14 Pa. At each CO_2_ level, the leaves were allowed to adjust for at least 30 min or until stable values of *A*_net_ and *g*_sw_ were achieved. Data for isotopologs of CO_2_ and H_2_O and physiological parameters (*A*_net_, *g*_sw_, *C*_i_, TE_i_) were collected and averaged over the next 20–30 min for each CO_2_ level (*c*. 10–15 cycles of TDLAS) with the Li6400XT set to log data only when the TDLAS analysed the sample line. At each CO_2_ level, three biological replicates were measured for the species eari and five biological replicates were measured for species' pvir and eser. For the remaining 13 species, measurements were conducted on four biological replicates. After the completion of above measurements, lights were tuned off and the leaves were allowed to stabilise for 15 min before logging the rates of dark respiration (*R*_n_, μmol CO_2_ m^−2^ s^−1^). Mesophyll conductance was estimated for each species at each of the four *p*CO_2_ levels using the Ogee *et al*. (2018) method. Key input parameters used in calculation of isotope parameters and estimation of *g*_m_ are given in Supporting Information Table S1. Further details of equations and calculations of fractionation factors can be found in Ogee *et al*. (2018); Ubierna *et al*. (2017). While estimating *g*_m_, we assumed that ϕr or the fraction of respired CO_2_ not produced in the bundle sheath cells of C_4_ plants = 0.5 (von Caemmerer, 2000) and analysed the impacts of changing ϕr values (0–1) on the calculation of *g*_m_ (Fig. S1). We also performed a sensitivity analysis of *g*_m_ to changes in leaf temperature (ranging from 23 to 28°C) (Fig. S2). For C_4_ plants, photorespiration was assumed to be negligible and therefore not accounted for while estimating *g*_m_. While estimating *g*_m_ at different *p*CO_2_ (34, 27, 20 and 14 Pa), it was assumed that the pH of the mesophyll cytosol was constant due to the small micromolar shifts in dissolved CO_2_ at different *p*CO_2_ (DiMario *et al*., 2018). We assumed that the day respiration (Vr, μmol CO_2_ m^−2^ s^−1^) was equal to the dark respiration (*R*_n_) for the C_4_ species.

### Measurement of anatomical traits and habitat mean annual precipitation

Light and electron microscopy techniques were used to measure important structural and anatomical traits such as adaxial stomatal density (SD_ada_, number mm^−2^), abaxial stomatal density (SD_aba_, number mm^−2^), SR (unitless) expressed as ratio of SD_ada_ : SD_aba_, leaf thickness (μm), mesophyll surface area exposed to intercellular air spaces (S_mes_, μm^2^ μm^−2^) and *T*_CW_ (μm). The details of sample preparation for microscopy and measurements are presented in Pathare *et al*. (2020a) and in the Methods S1. Values for mean annual precipitation (MAP) for habitats where the C_4_ grasses commonly occur were obtained as indicated in Pathare *et al*. (2020b).

### Enzyme assays and measurement of leaf nitrogen content

Immediately following gas exchange measurements, leaf samples were taken from the same leaf and frozen in liquid nitrogen for enzyme assays. Measurements of CA, phosphoenolpyruvate carboxylase (PEPC, μmol m^−2^ s^−1^) and ribulose‐1,5‐bisphosphate carboxylase/oxygenase (Rubisco, μmol m^−2^ s^−1^) activities were performed at 25°C, as described previously (Sharwood *et al*., 2016; Sonawane & Cousins, 2019; Pathare *et al*., 2020a). Carbonic anhydrase activities were expressed as the first‐order rate constant (*k*_CA_, μmol m^−2^ s^−1^ Pa^−1^). In addition to enzyme activities, PEPC affinity for HCO^−^_3_ (*K*_m_, μM HCO_3_^−1^) values were derived using a membrane‐inlet mass spectrometer. *K*_m_ values have been published in DiMario *et al*. (2021). For measuring leaf nitrogen content, leaf samples were taken from the same leaf on which gas exchange measurements were performed. Samples were dried in a hot air oven at 60°C for 72 h. Leaf nitrogen content was measured using a Eurovector elemental analyser and expressed on a leaf area basis (N_area_, g m^−2^).

### Estimating CO_2_ responses of physiological traits (*g*_m_, *g*_sw_, *A*_net_ and TE_i_ )

Changes in *g*_m_ in response to decrease in *p*CO_2_ (from 34 to 14 Pa) were analysed using two different methods. First, the percentage change in *g*_m_ in response to a decrease in *p*CO_2_ (from 34 to 14 Pa) was calculated for each biological replicate for the 16 species as follows:

$$\left(\text{trait value}\;\mathrm{at}\;14\;\mathrm{Pa}\;-\;\text{trait value}\;\mathrm{at}\;34\;\mathrm{Pa}\right)\times 100/\left(\text{trait value}\;\mathrm{at}\;34\;\mathrm{Pa}\right)$$

Similar to *g*_m_, we also calculated percentage change in *A*_net_, *g*_sw_, *C*_i_ and TE_i_ in response to the decrease in *p*CO_2_ (from 34 to 14 Pa). *p*CO_2_ levels of 34 and 14 Pa were chosen, as these two *p*CO_2_ levels were the highest and lowest levels, respectively, used in current study. Relationships of percentage change in physiological traits with key leaf anatomical and biochemical traits are given in the Figs S3–S8.

**Fig. 1:**
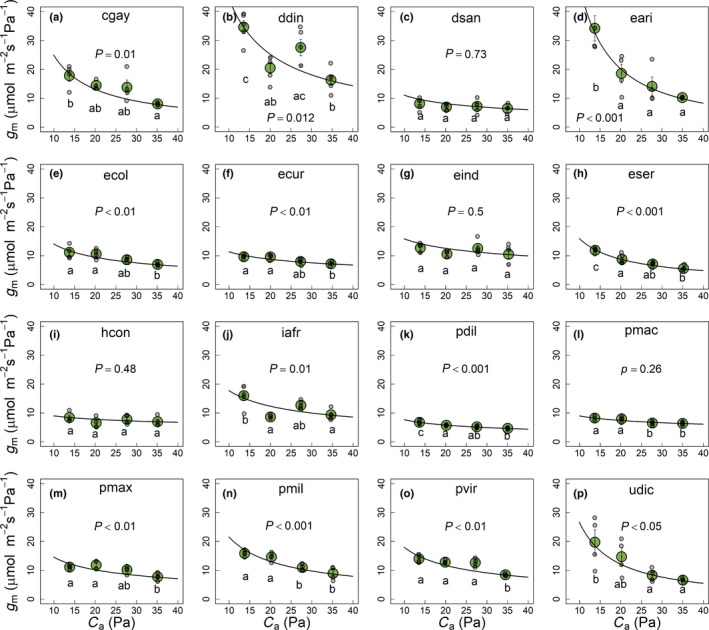
Response of mesophyll conductance (*g*_m_) to changes in the partial pressure of CO_2_ (*p*CO_2_) inside the leaf chamber (*C*_a_) in 16 diverse C_4_ grasses. Data for each of the species are shown separately from panels (a) to (p) along with the CO_2_ response of *g*_m_ (black solid line) modelled using the equation, *g*_m_ = *a* × (34/*C*_a_)^*b*^, where coefficient ‘*a*’ is the value of *g*_m_ at 34 Pa *p*CO_2_ and coefficient ‘*b*’ is the rate of change in *g*_m_ with change in *C*_a_. Mean ± SE values for the model constants (*a* and *b*) for each species are shown in Supporting Information Table S3. Measurements were performed at constant light (photosynthetic photon flux density (PPFD) = 1200 μmol m^−2^ s^−1^) and leaf temperature (25°C). Values in each panel represent the mean ± SE (green colour) with *n* = 3–6. Grey points indicate the replicate values for each species and CO_2_ level. Response of *g*_m_ to changes in *p*CO_2_ for each species is plotted in separate panel of Fig. 1. Species code has been indicated as the first letter of the genus and first three letters of the species (please refer to Table 1 for full names of species). *P‐*values from one‐way ANOVA along with Tukey's letters are shown.

**Fig. 2:**
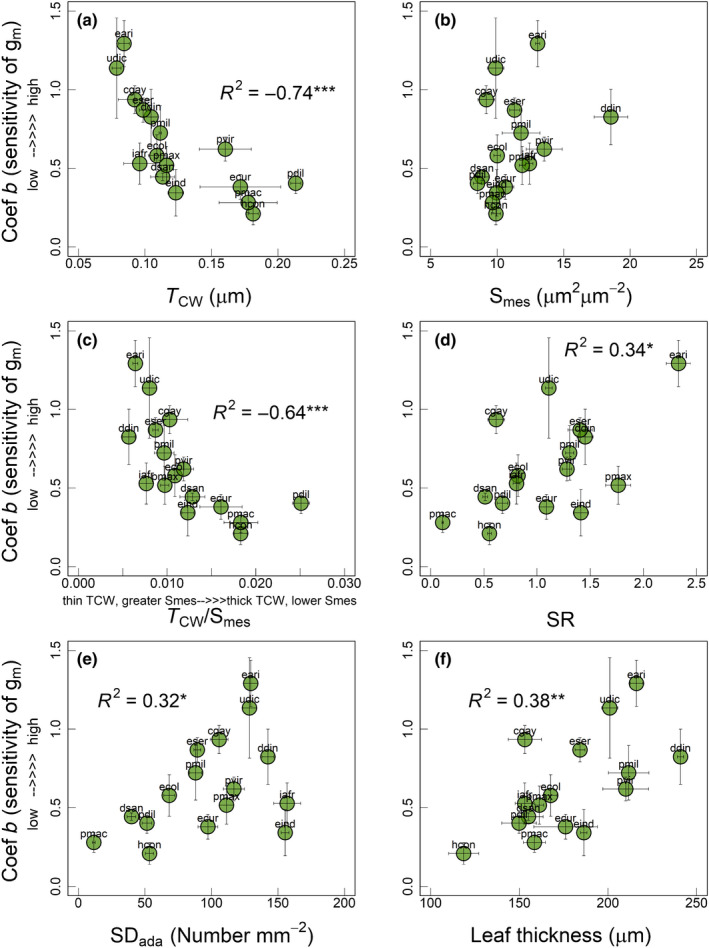
Relationship of coefficient *b* (or sensitivity of *g*_m_, unitless) with (a) mesophyll cell wall thickness (*T*_CW_), (b) mesophyll surface area exposed to intercellular air spaces (S_mes_), (c) ratio of *T*_CW_ : S_mes_, (d) stomatal ratio (SR), (e) stomatal density adaxial (SD_ada_) and (f) leaf thickness among the 16 C_4_ grasses measured in current study. Linear models were used for deriving regression coefficients (*R* ^2^) in all panels, except panels (a) and (c) for which we used a polynomial model. Significance of *R* ^2^: *, *P* ≤ 0.05; **, *P* ≤ 0.01; ***, *P* ≤ 0.001. Each circle represents the mean ± SE value for each species (*n* = 3–6). Species names are indicated by the codes given in Table 1.

**Fig. 3:**
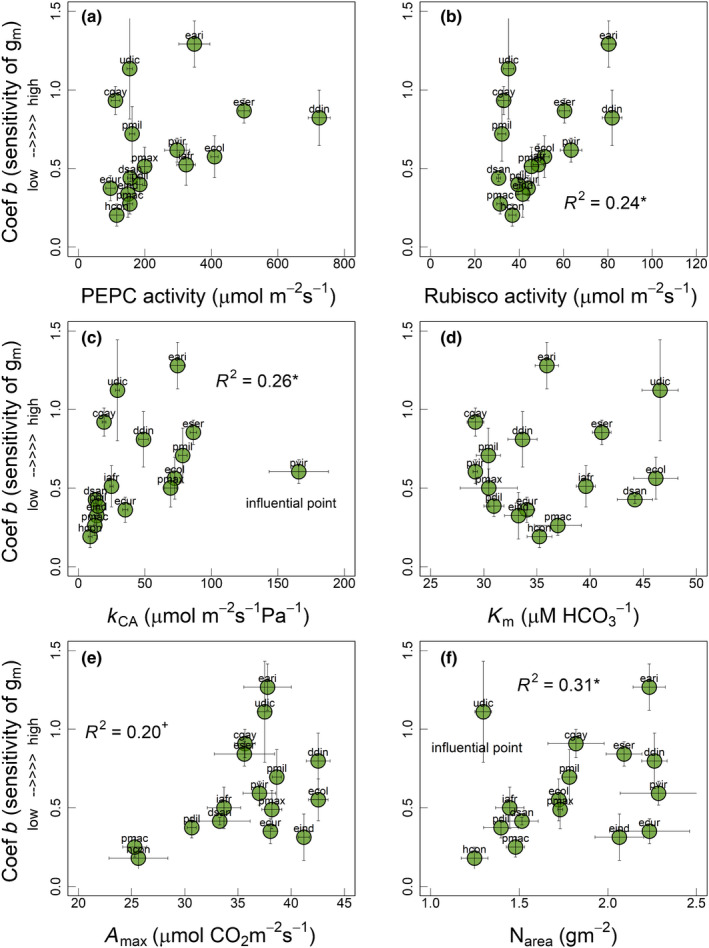
Relationship of coefficient *b* (or sensitivity of *g*_m_, unitless) with (a) phosphoenolpyruvate carboxylase (PEPC) activity, (b) Rubisco activity, (c) carbonic anhydrase (CA) activity expressed as *k*_CA_, (d) PEPC affinity for HCO_3_^−^ (*K*_m_), (e) maximum photosynthetic capacity (*A*_max_) and (f) leaf N content (N_area_) among the 16 C_4_ grasses measured in current study. Linear models were used for deriving regression coefficients (*R* ^2^) in all panels. In panel (c) *R* ^2^ = 0.22^+^ after removing influential species pvir. Significance of *R* ^2^: +, marginally significant; *, *P* ≤ 0.05. Each circle represents the mean ± SE value for each species (*n* = 3–6). Species names are indicated by codes given in Table 1.

**Fig. 4:**
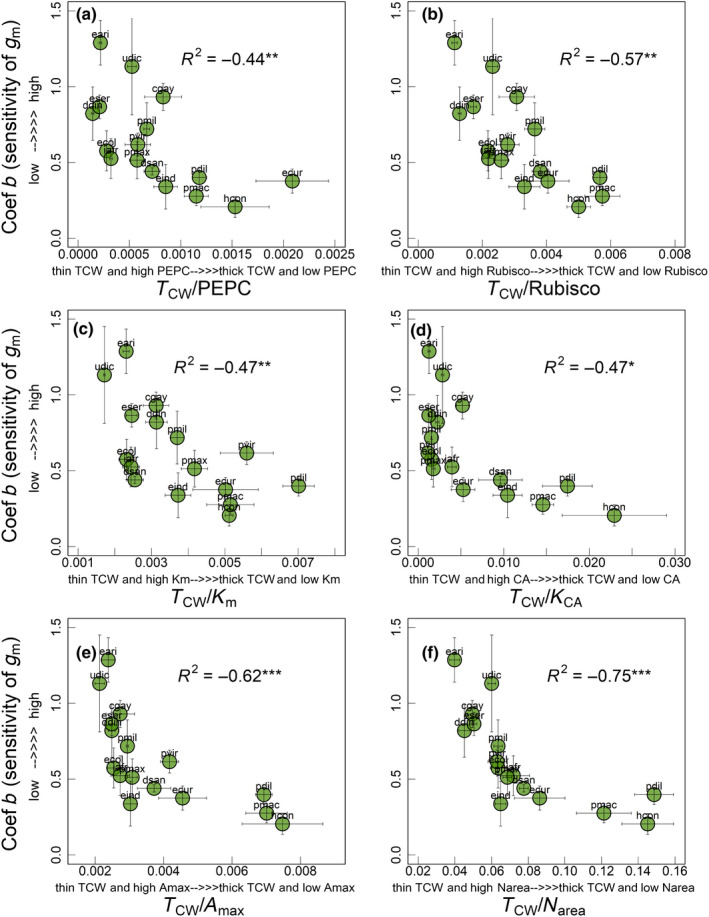
Relationship of coefficient *b* (or sensitivity of *g*_m_, unitless) with ratio of mesophyll cell wall thickness (*T*_CW_) to (a) phosphoenolpyruvate carboxylase (PEPC) activity (b) Rubisco activity (c) PEPC affinity for HCO_3_^−^ (*K*_m_), (d) carbonic anhydrase (CA) activity expressed as *k*_CA_, (e) maximum photosynthetic rates (*A*_max_) and (f) leaf N content (N_area_) among the 16 C_4_ grasses measured in current study. Polynomial models were used to derive regression coefficients (*R* ^2^) in all panels. Significance of *R* ^2^: *, *P* ≤ 0.05; **, *P* ≤ 0.01; ***, *P* ≤ 0.001. Each circle represents the mean ± SE value for each species (*n* = 3–6). Species names are indicated by codes given in Table 1.

In a second method, we used an equation (*y* = *a* × (34/CO_2_)^*b*^) to model the changes in *g*_m_ in response to changes in CO_2_ (here *C*_a_). In the equation, *y* indicates the *g*_m_, coefficient *a* (μmol m^−2^ s^−1^ Pa^−1^) is the value of *g*_m_ at 34 Pa *p*CO_2_, CO_2_ indicates the *p*CO_2_ inside the leaf chamber (*C*_a_) and coefficient *b* (unitless) indicates the sensitivity of *g*_m_ to changes in *C*_a_. For further analysis (Figs 2, 3, 4 please refer to later paragraphs), we considered coefficient *b* as a proxy for the CO_2_ response of *g*_m_, with a relatively greater value of *b* indicating a greater increase in *g*_m_ with a decrease in *C*_a_. Mean ± SE values for model coefficients *a* and *b* for the 16 C_4_ grasses are given in Table S3 (please refer to later paragraphs). Average values for three biochemical subtypes (NAD‐ME, NADP‐ME and PCK) are also included in Table S3. Fig. 1 shows the relationship of *g*_m_ and *C*_a_ for 16 C_4_ grasses along with the model line, whereas Fig. S9 shows relationship of *g*_m_ and *C*_a_ along with the model line for the three C_4_ subtypes.

### Statistical analyses

All statistical analyses were performed using R software (v.4.1.0, R Foundation for Statistical Computing, Vienna, Austria). Normality and equal variances were tested and, when necessary, square root or log transformations were used to improve the data homoscedasticity (Zar, 2007). One‐way ANOVA with *post‐hoc* Tukey's test was used to examine differences in leaf‐level anatomical and biochemical traits among the 16 diverse C_4_ grasses. Results for *post‐hoc* Tukey's test are given in Table 1. Results of one‐way ANOVA for traits used in the current study are given in Table S2. For key physiological traits such as *g*_m_, ∆^18^O, *A*_net_, *g*_sw_, *C*_i_ and TE_i_, two‐way ANOVA was performed with species and *p*CO_2_ as the main effects using the *aov* function in R (R Core Team, 2018). Results of two‐way ANOVA are given in Table 2 and mean values are presented in Figs S1–S6. For the ANOVAs, *P*‐values ≤ 0.05 were considered as statistically significant. We grouped the species into biochemical subtypes and analysed whether CO_2_ responses of *g*_m_ varied among the subtypes (Table S3; Fig. S9).

**Table 2:** Summary of two‐way ANOVA *P‐*values and *F*‐values along with numerator degree of freedom (*df*) for the physiological traits measured on 16 diverse C_4_ grasses at four different *p*CO_2_.

Traits	Species (*df* = 15)		CO_2_ (*df* = 3)		Species × CO_2_ (*df* = 45)	
	*P*‐value	*F*‐value	*P*‐value	*F*‐value	*P*‐value	*F*‐value
*g*_m_	**< 0.001**	39.18	**< 0.001**	64.85	**< 0.001**	2.54
∆^18^O	**< 0.001**	5.34	**< 0.001**	74.92	**0.04**	1.46
*g*_sw_	**< 0.001**	37.64	**< 0.001**	228.39	0.32	1.10
*A*_net_	**< 0.001**	63.20	**< 0.001**	201.63	0.96	0.64
*C*_i_	**< 0.001**	12.44	**< 0.001**	370.49	0.12	1.29
TE_i_	**< 0.001**	22.29	**< 0.001**	2764.65	0.16	1.24

Mean values for these physiological traits list below are presented in Supporting Information Figs S1–S5. Statistically significant *P*‐values (≤ 0.05) are highlighted in bold. Mesophyll conductance to CO_2_ (*g*_m_; estimated using the Ogee *et al*., 2018 method), leaf net C^18^O^16^O discrimination (∆^18^O) to changes in CO_2_ inside leaf, stomatal conductance to water (*g*_sw_), net CO_2_ assimilation rates (*A*_net_), leaf intercellular [CO_2_] (*C*_i_) and leaf‐level water‐use efficiency (TE_i_ = *A*_net_/*g*_sw_).

Regression analyses were performed to examine the relationship of coefficient *b* with the key anatomical and biochemical traits such as *T*_CW_, S_mes_, ratio of *T*_CW_ : S_mes_, SR, SD_ada_, leaf thickness, PEPC activity, Rubisco activity, *k*_CA_, *K*_m_, *A*_max_ and N_area_. To account for the combined effect of anatomy and biochemistry on the CO_2_ responses of *g*_m_, we derived ratios of *T*_CW_ to biochemical traits (activities of PEPC, Rubisco and CA, *K*_m_, *A*_max_ and N_area_). We examined the relationship between coefficient *b* and the ratio of *T*_CW_ to biochemical traits. Similarly, we also examined the relationships between percentage change in *g*_m_ and key anatomical and biochemical traits and the ratio of *T*_CW_ to the biochemical traits mentioned above. Both, coefficient *b* and percentage change in *g*_m_ showed similar relationships with anatomical and biochemical traits. In the main text we used coefficient *b* as a proxy for CO_2_ response of *g*_m_ (Figs 2, 3, 4). The relationship of percentage change in physiological traits with key leaf anatomical and biochemical traits are given in Figs S3–S8. For the regression analysis, *P*‐values ≤ 0.05 were considered as statistically significant. The function *outlierTest* from the R package car (Fox & Weisberg, 2019) was used to identify any potential influential points in the relationships. Influential data points (with Bonferroni *P*‐value ≤ 0.05) were removed while deriving regression statistics if required (Figs 3c,f, S3, S5, S7). To complement the regression analysis, we also performed a principal component analysis (PCA; Methods S2; Fig. S8; Table S4) with leaf traits, percentage changes in *g*_m_, *A*_net_, *g*_sw_ and TE_i_ and habitat MAP (R package factominer; Le *et al*., 2008).

## Results

### CO_2_ response of physiological traits

The 16 C_4_ grasses (Table 1) with previously demonstrated variation in leaf‐level anatomical traits and *g*_m_ (Pathare *et al*., 2020a,b) were chosen to explore the potential variation in the CO_2_ response of *g*_m_ and its relationship with leaf traits and TE_i_. Responses of *g*_m_ to changes in *C*_a_ and *C*_i_ are given in Figs 1 and S10, respectively. Responses of other physiological traits (∆^18^O, *g*_sw_, *A*_net_, *C*_i_ and TE_i_) to changes in *C*_a_ are given in Figs S11–S15. Species differed significantly in the CO_2_ response of *g*_m_ (Figs 1, S10) and *g*_sw_ (Fig. S12). In general, across the 16 C_4_ grasses, *g*_m_ increased as *C*_a_ and *C*_i_ decreased with percentage increase at lowest *C*_a_, ranging from +13% to +250%. The increase in *g*_m_ was statistically significant in 12 out of 16 species. As with *g*_m_, *g*_sw_ increased with decreasing *C*_a_ (Fig. S12). However, unlike *g*_m_, the magnitude of increase in *g*_sw_ was lower. Specifically, the percentage increase in *g*_sw_ ranged from *c*. 40–80% when *C*_a_ decreased from 34 to 14 Pa. Alternatively, both *A*_net_ and TE_i_ decreased with decreases in *C*_a_ (Figs S13, S15). Particularly, *A*_net_ decreased by *c*. 23–40% and TE_i_ decreased by *c*. 53–64%.

### Variation in leaf anatomical and biochemical traits

The results of one‐way ANOVA suggested that the 16 C_4_ grasses varied significantly (*P <* 0.001; Table 1) in all the leaf‐level anatomical and biochemical traits. *T*_CW_ showed a significant 2.6‐fold variation with values ranging from *c*. 0.08 to 0.21 μm. S_mes_ showed a 2.4‐fold variation with values ranging from *c*. 8.5 to 19 μm^2^ μm^−2^. The C_4_ grasses measured here also showed a highly significant variation in stomatal traits such as SR and SD_ada_ (*P <* 0.001; Tables 1, S2). SR varied by 4.7‐fold, with values ranging from 0.5 to 2.4, whereas SD_ada_ showed a 13‐fold variation with values ranging from 12 to 160 number mm^−2^. Leaf thickness showed a significant two‐fold variation with values ranging from 120 to 240 μm (*P <* 0.001; Tables 1, S2). Similar to the variation in anatomical traits mentioned above, the biochemical traits (that is leaf‐level activities of PEPC, Rubisco and *k*_CA_) also varied significantly among the 16 C_4_ grasses included in the current study (*P <* 0.001; Tables 1, S2). PEPC activities showed a 7.5‐fold variation with values from 96 to 721 μmol m^−2^ s^−1^. Rubisco activities showed a 2.6‐fold variation with values from 30 to 80 μmol m^−2^ s^−1^. *k*_CA_ showed an 18‐fold variation with values from 9 to 165 μmol m^−2^ s^−1^ Pa^−1^. PEPC affinity for HCO_3_^−^ (*K*_m_) showed a 1.6‐fold variation across the 16 C_4_ grasses with values ranging from *c*. 29 to 46 μM HCO_3_^−^ (*P <* 0.001; Tables 1, S2). There was a 1.7‐fold variation in maximum photosynthetic rates (*A*_max_) with values ranging from *c*. 25 to 42.5 μmol CO_2_ m^−2^ s^−1^ (*P <* 0.001; Table 1). N_area_ also varied significantly among the grasses (*P <* 0.001; Table 1) with values ranging from 1.24 to 2.26 g m^−2^.

### Relationships of CO_2_ response of *g*_m_ with anatomical and biochemical traits

We used two proxies to account for the changes in *g*_m_ in response to change in *C*_a_. First, percentage change in *g*_m_ in response to decrease in *C*_a_ (from 34 to 14 Pa) (Figs S3–S8) and second, the coefficient *b* derived from an equation used to model the changes in *g*_m_ in response to changes in *C*_a_. In the main text, we used coefficient *b* as a proxy for CO_2_ response of *g*_m_, (Figs 2, 3, 4). Coefficient *b* showed a strong negative relationship with *T*_CW_ (*R* ^2^ = −0.74, *P* < 0.001; Fig. 2a) and ratio of *T*_CW_ : S_mes_ (*R* ^2^ = −0.64, *P* < 0.001; Fig. 2c) and a nonsignificant positive relationship with S_mes_ (*R* ^2^ = 0.14, *P* = 0.11; Fig. 2b). In addition, coefficient *b* showed a significant positive relationship with SR (*R* ^2^ = 0.34, *P* < 0.05; Fig. 2d), SD_ada_ (*R* ^2^ = 0.32, *P* < 0.01; Fig. 2e) and leaf thickness (*R* ^2^ = 0.38, *P* < 0.01; Fig. 2f). Coefficient *b* also showed a nonsignificant positive relationship with biochemical traits such as PEPC activity (*R* ^2^ = 0.19, *P* = 0.12; Fig. 3a) and *A*_max_ (*R* ^2^ = 0.20, *P* = 0.08; Fig. 3e) and a significant positive relationship with Rubisco activity (*R* ^2^ = 0.24, *P* < 0.05; Fig. 3b). However, coefficient *b* did not show a statistically significant relationship with *K*_m_ (Fig. 3c,d). Coefficient *b* showed significant positive relationships with *k*_CA_ (*R* ^2^ = 0.26, *P* < 0.05; Fig. 3c) and N_area_ (*R* ^2^ = 0.31, *P* < 0.05; Fig. 3f) after removing the influential species (pvir and udic, respectively).

To account for the combined effects of leaf anatomy (specifically *T*_CW_ that acts as a resistance in series to CO_2_ diffusion) and biochemistry (that can have a facilitating effect) on CO_2_ response of C_4_–*g*_m_, we derived the ratio of *T*_CW_ with PEPC activity, Rubisco activity, *k*_CA_, *K*_m_, *A*_max_ and N_area_ and investigated the relationship between coefficient *b* and these ratios (Fig. 4). Coefficient *b* showed a significant negative relationship with *T*_CW_/PEPC (*R* ^2^ = −0.44, *P* < 0.01; Fig. 4a), *T*_CW_/Rubisco (*R* ^2^ = −0.57, *P* < 0.01; Fig. 4b), *T*_CW_/*K*_m_ (*R* ^2^ = −0.47, *P* < 0.01; Fig. 4c), *T*_CW_/*k*_CA_ (*R* ^2^ = −0.47, *P* < 0.05; Fig. 4d), *T*_CW_/*A*_max_ (*R* ^2^ = −0.62, *P* < 0.001; Fig. 4e) and *T*_CW_/N_area_ (*R* ^2^ = −0.75, *P* < 0.001; Fig. 4f). In summary, C_4_ grasses with thinner cell walls combined with greater S_mes_, N_area_, *A*_max_ and activities of key enzymes were able to achieve greater increases in *g*_m_ at lower *C*_a_. Similar results were observed when we plotted percentage change in *g*_m_ against key anatomical and biochemical traits mentioned above (Figs S3–S8).

### Relationship of CO_2_ response of *g*_m_ with CO_2_ response of TE_i_ , *g*_sw_ and *A*_net_

We investigated the impacts of changes in *g*_m_ with *C*_a_ on corresponding changes in TE_i_, *g*_sw_ and *A*_net_ (Fig. S7). For this we used percentage change in the trait's value when *C*_a_ decreased from 34 to 14 Pa. As *g*_m_ and *g*_sw_ increase with decreases in *C*_a_, percentage change for both conductances is reported in the manuscript as percentage increase. Alternatively, *A*_net_ and TE_i_ decrease with decreases in CO_2_S, therefore the percentage change for these two traits have been reported in the manuscript as percentage decrease. Percentage change in TE_i_ showed a strong negative correlation with percentage change in *g*_m_ (*R* ^2^ = −0.54, *P <* 0.001; Fig. S7a). Particularly, C_4_ grasses that were able to achieve a greater increase in *g*_m_ at lower *C*_a_ showed a lesser decrease in TE_i_ (indicated by a less negative value for TE_i_ in Fig. S11a). In addition, percentage change in *g*_sw_ showed a negative relationship with percentage change in *g*_m_ (*R* ^2^ = −0.28, *P <* 0.05; Fig. S7b), that is species that showed a greater increase in *g*_m_ at lower *C*_a_ also showed a lesser increase in *g*_sw_. A significant relationship was not observed between percentage change in *g*_m_ and *A*_net_ (Fig. S7c).

### CO_2_ response of *g*_m_ in the C_4_ biochemical subtypes

We also analysed the CO_2_ responses of *g*_m_ among the subtypes (Table S3; Fig. S9). NADP‐ME (coefficient *b* = 0.618) and PCK (coefficient *b* = 0.0.73) subtypes showed 20% and 43% greater sensitivity of *g*_m_ to *C*_a_ respectively compared with NAD‐ME (coefficient *b* = 0.512). However, there were no statistically significant differences in coefficient *b* among the subtypes (Fig. S9e), which could be due to the low replication. With only seven NADP‐ME, five PCK and four NAD‐ME replicate species our study does not provide the statistical power to discuss subtype effects. Therefore, here we focus on species‐level differences.

## Discussion

In general C_3_–*g*_m_ increases under short‐term decreases in *p*CO_2_ (Flexas *et al*., 2007; Bunce, 2010; Douthe *et al*., 2011), although not always (von Caemmerer & Evans, 1991; Loreto *et al*., 1992; Tazoe *et al*., 2009). The main candidates suggested to affect the CO_2_ response of C_3_–*g*_m_ include chloroplast movement and therefore changes in *S*_c_, changes in activities of CA (Evans *et al*., 2009; Momayyezi & Guy, 2017) and the facilitation effect of CO_2_‐permeable aquaporins (Uehlein *et al*., 2008; Flexas *et al*., 2012; Kaldenhoff, 2012). However, the C_3_–*g*_m_ response to *p*CO_2_ may also result from systematic errors associated with the use of methods such as gas exchange, chlorophyll fluorescence and discrimination against ^13^C or the use of oversimplified models (Pons *et al*., 2009; Yin & Struik, 2009; Tholen & Zhu, 2011; Gu & Sun, 2014). For instance, the apparent response of C_3_–*g*_m_ to short‐term changes in *p*CO_2_ has partly been attributed to changes in photorespiratory and nonphotorespiratory release of CO_2_ (or day respiration) to the total CO_2_ pool in the leaf, particularly under low *p*CO_2_. Ignoring these CO_2_ pools while estimating C_3_–*g*_m_ also overlooks the effects of spatial distribution of mitochondria and chloroplast on pathlength of CO_2_ movement. Inaccurate measurements of day respiration or estimates of the Rubisco fractionation factor in the ∆^13^C method and measurements conducted at low [O_2_] have also been suggested as potential sources of artefacts in determining the C_3_–*g*_m_ response to *p*CO_2_ (Pons *et al*., 2009; Gu & Sun, 2014).

Alternatively, in C_4_ species, the photorespiratory release of CO_2_ is relatively low and may not contribute significantly to estimates of *g*_m_, even at low *p*CO_2_ (Cousins *et al*., 2020). Also, in current study we estimated *g*_m_ in diverse C_4_ grasses by using a method based on modelling of ∆^18^O that considers the contribution of respiratory fluxes (Ogee *et al*., 2018). Despite some uncertainties associated with the day respiration or water isotope gradient between mesophyll and bundle sheath cells, the Ogee *et al*. (2018) method provides robust estimates of C_4_–*g*_m_. Also, comparing many diverse C_4_ species, as done in the current study, using a method that is not subject to the same limitations as those previously used for C_3_ species is a reasonable approach to identify the CO_2_ responses of C_4_–*g*_m_. Here, we discuss the CO_2_ response of *g*_m_ for 16 diverse C_4_ grasses and its relationships with leaf anatomical and biochemical traits.

### CO_2_ response of *g*_m_ varied among diverse C_4_ grasses

Very few studies investigating the CO_2_ response of C_4_–*g*_m_, primarily maize and *Setaria viridis* (Osborn *et al*., 2017; Kolbe & Cousins, 2018; Ubierna *et al*., 2018), have reported an increase in C_4_–*g*_m_ with a decrease in *p*CO_2_. Similarly, we demonstrated that *g*_m_ increases with a decrease in *p*CO_2_ across the 16 diverse C_4_ grasses. However, the magnitude of the increase in *g*_m_ varied greatly across the 16 C_4_ grasses (+13% to +250%; Fig. 1). In the following sections we discuss the potential factors that could explain this variability in the CO_2_ response of C_4_–*g*_m_.

### CO_2_ response of C_4_ –*g*_m_ is related with *T*_CW,_ S_mes_ and photosynthetic capacity

*g*_m_ in C_3_ and C_4_ species is constrained by several anatomical and biochemical parameters such as *T*_CW_, S_mes_, S_c_, CA activities and aquaporins (Cousins *et al*., 2020; Evans, 2021). However, the potential implication of leaf anatomy and biochemistry for the CO_2_ response of *g*_m_ has not been studied for C_4_ species. Most of the anatomical parameters remain unchanged under short‐term changes in environmental conditions such as *p*CO_2_ (Evans *et al*., 2009; Terashima *et al*., 2011). Therefore, the only anatomical trait suggested to influence the CO_2_ response of *g*_m_ is chloroplast movement that can affect *g*_m_ by changing S_c_ (Terashima *et al*., 2006; Tholen *et al*., 2008; Carriqui *et al*., 2018). However, chloroplast movement is unlikely to explain the variability of the CO_2_ response of C_4_–*g*_m_ observed in the current study, because S_mes_ and not S_c_ is a more accurate determinant of C_4_–*g*_m_, in which a greater S_mes_ results in greater *g*_m_ in C_4_ species (Barbour *et al*., 2016; Pathare *et al*., 2020a). In contrast with S_c_, S_mes_ should remain unchanged under short‐term changes in *p*CO_2_. Therefore, although we observed a nonsignificant positive relationship between the CO_2_ response of C_4_–*g*_m_ and S_mes_ (Fig. 2b), S_mes_ may provide only a partial explanation for this variable response.

*T*_CW_ could account for > 50% of the total resistance to CO_2_ diffusion inside leaves (Evans *et al*., 2009). Therefore, we further investigated the influence of *T*_CW_ on the variability of the CO_2_ response of C_4_–*g*_m_. In general, C_3_ species with relatively lower *T*_CW_ showed greater *g*_m_ (Onoda *et al*., 2017; Veromann‐Jürgenson *et al*., 2017; Evans, 2021). We did not observe a strong relationship between *g*_m_ and *T*_CW_ for the C_4_ grasses under ambient CO_2_ levels (34 Pa) (Pathare *et al*., 2020a). However, in the current study, the CO_2_ response of C_4_–*g*_m_ was related to *T*_CW_ (Fig. 2a). The CO_2_ response of C_4_–*g*_m_ also showed a strong relationship with *T*_CW_ after accounting for S_mes_ (as *T*_CW_/S_mes_; Fig. 2c). Particularly, C_4_ grasses with relatively lower *T*_CW_ and greater S_mes_, showed a greater increase in *g*_m_ at low *p*CO_2_. It is unclear why the *g*_m_ of C_4_ grasses with lower *T*_CW_ is more responsive to changes in *p*CO_2_. As for S_mes_, *T*_CW_ is unlikely to change under short‐term changes in *p*CO_2_ and may not be the sole reason for the observed variable CO_2_ response of C_4_–*g*_m_. However, *T*_CW_ may still provide a partial explanation for variability in the CO_2_ response of C_4_–*g*_m_. Resistances to CO_2_ diffusion through the liquid phase (comprised of apoplastic and cellular components from mesophyll cell wall to site of carboxylation) are greater compared with the gaseous phase (Evans *et al*., 2009; Flexas *et al*., 2021). CO_2_ must dissolve in the water‐filled pores of the mesophyll cell walls and then diffuse to the plasma membrane and eventually to the site of CO_2_ fixation. Because cell walls represent a significant proportion of liquid phase resistance, C_4_ species with a lower *T*_CW_ may have the potential to achieve a greater change in *g*_m_ in response to changing *p*CO_2_. However, the influence of lower *T*_CW_ on the CO_2_ response of C_4_–*g*_m_ may have also been augmented by greater S_mes_, the facilitation effect of CO_2_ transporting aquaporins and leaf N content and proteins of photosynthetic machinery that determine drawdown and fixation of CO_2_ and therefore photosynthetic capacity (Parkhurst, 1994; Wright *et al*., 2004; Evans *et al*., 2009; Xiong & Flexas, 2021).

The role of CO_2_‐permeable aquaporins in enhancing *g*_m_ has been well characterised in C_3_ species (Uehlein *et al*., 2008). Only recently, it has been demonstrated that overexpressing a CO_2_‐permeable aquaporin in plasma membranes of *Setaria viridis* (C_4_ grass) can enhance C_4_–*g*_m_ (Ermakova *et al*., 2021). We did not investigate the role of aquaporins in affecting the CO_2_ response of C_4_–*g*_m_. However, we observed a greater CO_2_ response of *g*_m_ in C_4_ grasses with relatively lower *T*_CW_. This indicates that *g*_m_ in plants with lower *T*_CW_ may be more influenced by aquaporins (Evans, 2021). There is still a need to further investigate the role of aquaporins in the variable CO_2_ response of C_4_–*g*_m_.

Here, we investigated the relationship between the CO_2_ response of C_4_–*g*_m_ and photosynthetic capacity as indicated by *A*_max_, N_area_ and activities of key C_4_ photosynthetic enzymes (PEPC, CA and Rubisco). C_4_ species with a greater CO_2_ response of C_4_–*g*_m_ tended to show greater activities of PEPC and Rubisco and greater *A*_max_ and N_area_. Also, the CO_2_ response of C_4_–*g*_m_ was strongly related to the ratio of *T*_CW_/photosynthetic capacity traits (Fig. 4), in which a greater CO_2_ response of C_4_–*g*_m_ was evident in species with lower *T*_CW_ and greater photosynthetic capacity. This suggests that lower *T*_CW_ may have decreased the resistance to the movement of CO_2_ into the mesophyll cells (Evans, 2021; Flexas *et al*., 2021), whereas the greater photosynthetic capacity may have increased the demand for CO_2_, resulting in greater drawdown of CO_2_ and the necessity of maintaining a greater CO_2_ supply through an increase in *g*_m_ at low *p*CO_2_ (Wright *et al*., 2004; Evans *et al*., 2009). Furthermore, the enzyme CA catalyses the conversion of CO_2_/HCO_3_^−^ in the cytosol and ensures sufficient HCO_3_^−^ substrate supply to PEPC (Studer *et al*., 2014; DiMario *et al*., 2018). Because CO_2_ diffuses much faster in liquid phase in HCO_3_^−^ form, greater CA activities in combination with lower *T*_CW_ could have maintained a rapid supply of HCO_3_^−^ substrate to PEPC and further enhanced *g*_m_ at low *p*CO_2_ in some C_4_ grasses (Fig. 4d).

We also investigated the relationship between the CO_2_ response of C_4_–*g*_m_ and *K*_m_, a kinetic constant indicating PEPC affinity for HCO_3_^−^ (DiMario *et al*., 2021). In general, lower *K*_m_ values (or high affinity of PEPC for HCO_3_^−^) are expected to provide a selective advantage by maintaining high rates of C_4_ photosynthesis, particularly under conditions such as drought when CO_2_ availability is low due to restricted stomatal conductance. Here, we observed that *K*_m_ alone did not show a strong relationship with CO_2_ response of C_4_–*g*_m_ (Fig. 3d). However, after accounting for *T*_CW_, we observed that *T*_CW_/*K*_m_ showed a significant negative relationship with the CO_2_ response of C_4_–*g*_m_ (Fig. 4c), in which species with relatively lower *T*_CW_ and higher *K*_m_ (or lower affinity of PEPC for HCO_3_^−^) exhibited a greater CO_2_ response of *g*_m_. This contrasts with the general expectation and could be explained by the lower *T*_CW_ leading to higher *g*_m_ and with the corresponding greater CA activities leading to higher HCO_3_^−^ in mesophyll cells under low *C*_i_ conditions. The higher HCO_3_^−^ concentration in the mesophyll cells of species with lower *T*_CW_ and greater CA can reduce the selective pressure on PEPC for lower *K*_m_ values.

### Relationship of CO_2_ response of *g*_m_ with CO_2_ response of *g*_sw_ and TE_i_

Previously, we demonstrated that C_4_–*g*_m_ is positively related to TE_i_ under ambient *p*CO_2_ (Pathare *et al*., 2020a). Our current study further suggests that, for C_4_ grasses, *g*_m_ may also influence TE_i_ under short‐term changes in *p*CO_2_. In general, both *g*_m_ and g_sw_ increased (Figs 1, S12) and TE_i_ decreased at low *p*CO_2_ (Fig. S15). However, the magnitude of increase in *g*_m_ at low *p*CO_2_ was greater (values from 13% to 250%) compared with the increase in *g*_sw_ (values from 40% to 80%). Also, C_4_ grasses showing greatest increase in *g*_m_ at low *p*CO_2_ also showed the lowest increase in *g*_sw_ (Fig. S7b). Consequently, although TE_i_ decreased at low *p*CO_2_, the decrease was less in the species showing the greater CO_2_ response of *g*_m_ (Fig. S7a). These types of species with greater CO_2_ response of *g*_m_ may benefit in terms of maintaining TE_i_ under low CO_2_ conditions, such as drought, compared with species whose *g*_m_ was less responsive to changes in *p*CO_2_. Also, the PCA (Fig. S8) suggests that a greater CO_2_ response of *g*_m_ is generally observed in C_4_ grasses adapted to habitats with relatively low MAP.

### Conclusions

We demonstrated that C_4_–*g*_m_ increases with decreases in *p*CO_2_ and the magnitude of this increase in *g*_m_ varies greatly among the 16 diverse C_4_ grasses. Also, CO_2_ responses of C_4_–*g*_m_ is a composite trait that seems to be influenced by many leaf anatomical and biochemical parameters. The greatest increase in *g*_m_ at low *p*CO_2_ was observed in C_4_ grasses with lower *T*_CW_ along with greater S_mes_ and photosynthetic capacities. These C_4_ grasses with a greater CO_2_ response of *g*_m_ were also able to maintain their TE_i_ under low *p*CO_2_, which may be advantageous under low CO_2_ conditions such as drought. Our study advances the understanding of the CO_2_ response of *g*_m_ in diverse C_4_ species and identifies the key leaf anatomical and biochemical traits related to this response. This understanding is essential for improving C_4_ photosynthetic models (von Caemmerer, 2021) that ultimately feed the larger Earth system models (Rogers *et al*., 2017; Knauer *et al*., 2019a,b) and in attempts to improve water‐use efficiency of C_4_ crops through modification of *g*_m_ (von Caemmerer & Furbank, 2016).

## Competing interests

None declared.

## Author contributions

VSP and ABC designed the experiment. VSP, RJD and NK performed the measurements and analysed the data. VSP interpreted the data and led the writing with constructive inputs from RJD, NK and ABC.

## Supporting information


**Fig. S1** Sensitivity of mesophyll conductance (*g*_m_; estimated by Ogee *et al*., 2018 method) to changes in fraction of CO_2_ not produced in bundle sheath cells (ϕr).
**Fig. S2** Sensitivity of mesophyll conductance (*g*_m_; estimated by Ogee *et al*., 2018 method) to changes in leaf temperature (*T*_leaf_).
**Fig. S3** Relationship of model coefficient a (indicating value of *g*_m_ at 34 Pa *p*CO_2_) with coefficient *b* (sensitivity of *g*_m_ to *C*_a_; relatively lower *b* values indicate lower rate of change in *g*_m_ with *C*_a_) and relationship between coefficient *b* and percentage change in *g*_m_.
**Fig. S4** Relationship of percent increase in *g*_m_ with mesophyll cell wall thickness (*T*_CW_) mesophyll surface area exposed to intercellular air spaces (S_mes_) ratio of *T*_CW_ : S_mes_, stomatal ratio (SR), stomatal density adaxial (SD_ada_) and leaf thickness among the 16 C_4_ grasses measured in current study.
**Fig. S5** Relationship of percent increase in *g*_m_ with PEPC activity Rubisco activity CA activity expressed as *k*_CA_, PEPC affinity for HCO_3_^−^ (*K*_m_), maximum photosynthetic capacity (*A*_max_) and leaf N content (N_area_) among the 16 C_4_ grasses measured in current study.
**Fig. S6** Relationship of percent increase in *g*_m_ with ratio of mesophyll cell wall thickness (*T*_CW_) to PEPC activity Rubisco activity PEPC affinity for HCO_3_^−^ (*K*_m_), CA activity expressed as *k*_CA_, maximum photosynthetic rates (*A*_max_) and leaf N content (N_area_) among the 16 C_4_ grasses measured in current study.
**Fig. S7** Relationship of percent increase in *g*_m_ with percent decrease in leaf‐level water‐use efficiency (TE_i_) expressed as *A*_net_/*g*_sw_ (higher negative value indicates greater decrease in TE_i_), percent increase in stomatal conductance to water (*g*_sw_) and percent decrease in net photosynthetic rates (*A*_net_) (higher negative value indicates greater decrease in *A*_net_) for the 16 C_4_ grasses measured in current study.
**Fig. S8** PCA biplot showing major axes of variation in important leaf‐level anatomical and biochemical traits and percent change (increase or decrease) in response to CO_2_ in physiological traits such as *g*_m_, *A*_net_, *g*_sw_ and TE_i_ for the 16 diverse C_4_ grasses measured in current study.
**Fig. S9** Response of mesophyll conductance (*g*_m_) to changes in *p*CO_2_ inside leaf chamber (*C*_a_) in three C_4_ biochemical subtypes (NAD‐ME, NADP‐ME, PCK).
**Fig. S10** Response of mesophyll conductance to CO_2_ (*g*_m_) to changes in intercellular CO_2_ (*C*_i_) in 16 diverse C_4_ grasses measured in current study.
**Fig. S11** Response of leaf net C^18^O^16^O discrimination (∆^18^O) to changes in *p*CO_2_ inside leaf chamber (*C*_a_) in 16 diverse C_4_ grasses measured in current study.
**Fig. S12** Response of stomatal conductance to water (*g*_sw_) to changes in *p*CO_2_ inside leaf chamber (*C*_a_) 16 diverse C_4_ grasses measured in current study.
**Fig. S13** Response of net CO_2_ assimilation rates (*A*_net_) to changes in *p*CO_2_ inside leaf chamber (*C*_a_) in 16 diverse C_4_ grasses measured in current study.
**Fig. S14** Response of leaf intercellular CO_2_ concentration (*C*_i_) to changes in *p*CO_2_ inside leaf chamber (*C*_a_) in 16 diverse C_4_ grasses measured in current study.
**Fig. S15** Response of leaf‐level water‐use efficiency (TE_i_ = *A*_net_/*g*_sw_) to changes in *p*CO_2_ inside leaf chamber (*C*_a_) in 16 diverse C_4_ grasses measured in current study.
**Methods S1** Measurement of anatomical traits.
**Methods S2** Principal component analysis.
**Table S1** Key input parameters used in calculation of isotope parameters and estimation of mesophyll conductance (*g*_m_) for the 16 C_4_ grasses at four *p*CO_2_ levels (34, 27, 20 and 14 Pa) and a temperature of 25°C.
**Table S2** Results of one‐way ANOVA with species as main effects for all the leaf‐level anatomical and biochemical traits measured for 16 C_4_ grasses in current study.
**Table S3** C_4_ grasses used in current study along with their biochemical subtype and mean ± SE values for the equation (*g*_m_ = *a* × (34/*C*_a_)^*b*^) constants derived for the CO_2_ response of *g*_m_ in the 16 C_4_ grasses.
**Table S4** Component loadings for important leaf‐level traits determined on 16 C_4_ grasses.
Please note: Wiley Blackwell are not responsible for the content or functionality of any Supporting Information supplied by the authors. Any queries (other than missing material) should be directed to the *New Phytologist* Central Office.

## Data Availability

Data available on request from the authors.

## References

[nph18427-bib-0001] Barbour MM , Evans JR , Simonin KA , von Caemmerer S . 2016. Online CO_2_ and H_2_O oxygen isotope fractionation allows estimation of mesophyll conductance in C_4_ plants, and reveals that mesophyll conductance decreases as leaves age in both C_4_ and C_3_ plants. New Phytologist 210: 875–889. 26778088 10.1111/nph.13830

[nph18427-bib-0002] Barbour MM , Kaiser BN . 2016. The response of mesophyll conductance to nitrogen and water availability differs between wheat genotypes. Plant Science 251: 119–127. 27593470 10.1016/j.plantsci.2016.03.012

[nph18427-bib-0003] Bunce JA . 2010. Variable responses of mesophyll conductance to substomatal carbon dioxide concentration in common bean and soybean. Photosynthetica 48: 507–512.

[nph18427-bib-0004] von Caemmerer S . 2000. Biochemical models of leaf photosynthesis. Collingwood, Vic., Australia: CSIRO.

[nph18427-bib-0005] von Caemmerer S . 2021. Updating the steady‐state model of C_4_ photosynthesis. Journal of Experimental Botany 72: 6003–6017. 34173821 10.1093/jxb/erab266 PMC8411607

[nph18427-bib-0006] von Caemmerer S , Evans JR . 1991. Determination of the average partial pressure of CO_2_ in chloroplasts from leaves of several C_3_ plants. Functional Plant Biology 18: 287–305.

[nph18427-bib-0007] von Caemmerer S , Evans JR . 2015. Temperature responses of mesophyll conductance differ greatly between species. Plant, Cell & Environment 38: 629–637. 10.1111/pce.12449 25224884

[nph18427-bib-0008] von Caemmerer S , Furbank RT . 2016. Strategies for improving C_4_ photosynthesis. Current Opinion in Plant Biology 31: 125–134. 27127850 10.1016/j.pbi.2016.04.003

[nph18427-bib-0009] Cano FJ , Sharwood RE , Cousins AB , Ghannoum O . 2019. The role of leaf width and conductances to CO_2_ in determining water use efficiency in C_4_ grasses. New Phytologist 223: 1280–1295. 31087798 10.1111/nph.15920

[nph18427-bib-0010] Carriqui M , Douthe C , Molins A , Flexas J . 2018. Leaf anatomy does not explain apparent short‐term responses of mesophyll conductance to light and CO_2_ in tobacco. Physiologia Plantaram 165: 604–618. 10.1111/ppl.12755 29744895

[nph18427-bib-0011] Cousins AB , Mullendore DL , Sonawane BV . 2020. Recent developments in mesophyll conductance in C_3_, C_4_, and crassulacean acid metabolism plants. The Plant Journal 101: 816–830. 31960507 10.1111/tpj.14664

[nph18427-bib-0012] DiMario RJ , Kophs AN , Pathare VS , Schnable JC , Cousins AB . 2021. Kinetic variation in grass phospho*enol*pyruvate carboxylases provides opportunity to enhance C_4_ photosynthetic efficiency. The Plant Journal 105: 1677–1688. 33345397 10.1111/tpj.15141

[nph18427-bib-0013] DiMario RJ , Machingura MC , Waldrop GL , Moroney JV . 2018. The many types of carbonic anhydrases in photosynthetic organisms. Plant Science 268: 11–17. 29362079 10.1016/j.plantsci.2017.12.002

[nph18427-bib-0014] Douthe C , Dreyer E , Epron D , Warren CR . 2011. Mesophyll conductance to CO_2_, assessed from online TDL‐AS records of ^13^CO_2_ discrimination, displays small but significant short‐term responses to CO_2_ and irradiance in *Eucalyptus* seedlings. Journal of Experimental Botany 62: 5335–5346. 21841176 10.1093/jxb/err141 PMC3223034

[nph18427-bib-0015] Ellsworth PV , Ellsworth PZ , Koteyeva NK , Cousins AB . 2018. Cell wall properties in *Oryza sativa* influence mesophyll CO_2_ conductance. New Phytologist 219: 66–76. 29676468 10.1111/nph.15173

[nph18427-bib-0016] Ermakova M , Osborn H , Groszmann M , Bala S , McGaughey S , Byrt C , Alonso‐Cantabrana H , Tyerman S , Furbank RT , Sharwood RE *et al*. 2021. Expression of a CO_2_‐permeable aquaporin enhances mesophyll conductance in the C_4_ species *Setaria viridis* . *bioRxiv*. doi: 10.1101/2021.04.28.441895. PMC8648302 34842138

[nph18427-bib-0017] Evans JR . 2021. Mesophyll conductance: walls, membranes and spatial complexity. New Phytologist 229: 1864–1876. 33135193 10.1111/nph.16968

[nph18427-bib-0018] Evans JR , Kaldenhoff R , Genty B , Terashima I . 2009. Resistances along the CO_2_ diffusion pathway inside leaves. Journal of Experimental Botany 60: 2235–2248. 19395390 10.1093/jxb/erp117

[nph18427-bib-0019] Evans JR , von Caemmerer S . 1996. carbon dioxide diffusion inside leaves. Plant Physiology 110: 339–346. 12226185 10.1104/pp.110.2.339 PMC157726

[nph18427-bib-0020] Farquhar GD , Cernusak LA . 2012. Ternary effects on the gas exchange of isotopologues of carbon dioxide. Plant, Cell & Environment 35: 1221–1231. 10.1111/j.1365-3040.2012.02484.x 22292425

[nph18427-bib-0021] Fox J , Weisberg S . 2019. An R companion to applied regression, *3^rd^ edn* . Thousand Oaks, CA, USA: Sage.

[nph18427-bib-0022] Flexas J , Barbour MM , Brendel O , Cabrera HM , Carriquí M , Díaz‐Espejo A , Douthe C , Dreyer E , Ferrio JP , Gago J *et al*. 2012. Mesophyll diffusion conductance to CO_2_: an unappreciated central player in photosynthesis. Plant Science 193‐194: 70–84. 10.1016/j.plantsci.2012.05.009 22794920

[nph18427-bib-0023] Flexas J , Carriquí M , Coopman RE , Gago J , Galmés J , Martorell S , Morales F , Diaz‐Espejo A . 2014. Stomatal and mesophyll conductances to CO_2_ in different plant groups: underrated factors for predicting leaf photosynthesis responses to climate change? Plant Science 226: 41–48. 25113449 10.1016/j.plantsci.2014.06.011

[nph18427-bib-0024] Flexas J , Clemente‐Moreno MJ , Bota J , Brodribb TJ , Gago J , Mizokami Y , Nadal M , Perera‐Castro AV , Roig‐Oliver M , Sugiura D *et al*. 2021. Cell wall thickness and composition are involved in photosynthetic limitation. Journal of Experimental Botany 72: 3971–3986. 33780533 10.1093/jxb/erab144

[nph18427-bib-0025] Flexas J , Diaz‐Espejo A , GalmÉS J , Kaldenhoff R , Medrano H , Ribas‐Carbo M . 2007. Rapid variations of mesophyll conductance in response to changes in CO_2_ concentration around leaves. Plant, Cell & Environment 30: 1284–1298. 10.1111/j.1365-3040.2007.01700.x 17727418

[nph18427-bib-0026] Flexas J , Ribas‐CarbÓ M , Diaz‐Espejo A , GalmÉS J , Medrano H . 2008. Mesophyll conductance to CO_2_: current knowledge and future prospects. Plant, Cell & Environment 31: 602–621. 10.1111/j.1365-3040.2007.01757.x 17996013

[nph18427-bib-0027] Gillon JS , Yakir D . 2000. Internal conductance to CO_2_ diffusion and C^18^OO discrimination in C_3_ leaves. Plant Physiology 123: 201–214. 10806237 10.1104/pp.123.1.201 PMC58994

[nph18427-bib-0028] Grassi G , Magnani F . 2005. Stomatal, mesophyll conductance and biochemical limitations to photosynthesis as affected by drought and leaf ontogeny in ash and oak trees. Plant, Cell & Environment 28: 834–849.

[nph18427-bib-0029] Groszmann M , Osborn HL , Evans JR . 2017. Carbon dioxide and water transport through plant aquaporins. Plant, Cell & Environment 40: 938–961. 10.1111/pce.12844 27739588

[nph18427-bib-0030] Gu L , Sun Y . 2014. Artefactual responses of mesophyll conductance to CO_2_ and irradiance estimated with the variable J and online isotope discrimination methods. Plant, Cell & Environment 37: 1231–1249. 10.1111/pce.12232 24237289

[nph18427-bib-0031] Hassiotou F , Ludwig M , Renton M , Veneklaas EJ , Evans JR . 2009. Influence of leaf dry mass per area, CO_2_, and irradiance on mesophyll conductance in sclerophylls. Journal of Experimental Botany 60: 2303–2314. 19286919 10.1093/jxb/erp021

[nph18427-bib-0032] Huang X , Wang Z , Huang J , Peng S , Xiong D . 2021. Mesophyll conductance variability of rice aquaporin knockout lines at different growth stages and growing environments. The Plant Journal 107: 1503–1512. 34181799 10.1111/tpj.15397

[nph18427-bib-0033] Kaldenhoff R . 2012. Mechanisms underlying CO_2_ diffusion in leaves. Current Opinion in Plant Biology 15: 276–281. 22300606 10.1016/j.pbi.2012.01.011

[nph18427-bib-0034] Knauer J , Zaehle S , De Kauwe MG , Bahar NHA , Evans JR , Medlyn BE , Reichstein M , Werner C . 2019a. Effects of mesophyll conductance on vegetation responses to elevated CO_2_ concentrations in a land surface model. Global Change Biology 25: 1820–1838. 30809890 10.1111/gcb.14604 PMC6487956

[nph18427-bib-0035] Knauer J , Zaehle S , De Kauwe MG , Haverd V , Reichstein M , Sun Y . 2019b. Mesophyll conductance in land surface models: effects on photosynthesis and transpiration. The Plant Journal 101: 858–873. 31659806 10.1111/tpj.14587

[nph18427-bib-0036] Kolbe AR , Cousins AB . 2018. Mesophyll conductance in *Zea mays* responds transiently to CO_2_ availability: implications for transpiration efficiency in C_4_ crops. New Phytologist 217: 1463–1474. 29220090 10.1111/nph.14942

[nph18427-bib-0037] Kromdijk J , Głowacka K , Long SP . 2019. Photosynthetic efficiency and mesophyll conductance are unaffected in *Arabidopsis thaliana* aquaporin knock‐out lines. Journal of Experimental Botany 71: 318–329. 10.1093/jxb/erz442 31731291

[nph18427-bib-0038] Le S , Josse J , Husson F . 2008. factominer: an R package for multivariate analysis. Journal of Statistical Software 25: 1–18.

[nph18427-bib-0039] Loreto F , Harley PC , Di Marco G , Sharkey TD . 1992. Estimation of mesophyll conductance to CO_2_ flux by three different methods. Plant Physiology 98: 1437–1443. 16668812 10.1104/pp.98.4.1437 PMC1080369

[nph18427-bib-0040] Mizokami Y , Noguchi K , Kojima M , Sakakibara H , Terashima I . 2018. Effects of instantaneous and growth CO_2_ levels and abscisic acid on stomatal and mesophyll conductances. Plant, Cell & Environment 42: 1257–1269. 10.1111/pce.13484 30468514

[nph18427-bib-0041] Momayyezi M , Guy RD . 2017. Substantial role for carbonic anhydrase in latitudinal variation in mesophyll conductance of *Populus trichocarpa* Torr. & Gray. Plant, Cell & Environment 40: 138–149. 10.1111/pce.12851 27761902

[nph18427-bib-0042] Muir CD , Hangarter RP , Moyle LC , Davis PA . 2014. Morphological and anatomical determinants of mesophyll conductance in wild relatives of tomato (*Solanum* sect. *Lycopersicon*, sect. *Lycopersicoides*; Solanaceae). Plant, Cell & Environment 37: 1415–1426. 10.1111/pce.12245 24279358

[nph18427-bib-0043] Ogee J , Wingate L , Genty B . 2018. Mesophyll conductance from measurements of C^18^OO photosynthetic discrimination and carbonic anhydrase activity. Plant Physiology 178: 728–752. 30104255 10.1104/pp.17.01031 PMC6181052

[nph18427-bib-0044] Oguchi R , Hikosaka K , Hirose T . 2005. Leaf anatomy as a constraint for photosynthetic acclimation: differential responses in leaf anatomy to increasing growth irradiance among three deciduous trees. Plant, Cell & Environment 28: 916–927.

[nph18427-bib-0045] Onoda Y , Wright IJ , Evans JR , Hikosaka K , Kitajima K , Niinemets Ü , Poorter H , Tosens T , Westoby M . 2017. Physiological and structural tradeoffs underlying the leaf economics spectrum. New Phytologist 214: 1447–1463. 28295374 10.1111/nph.14496

[nph18427-bib-0046] Osborn HL , Alonso‐Cantabrana H , Sharwood RE , Covshoff S , Evans JR , Furbank RT , von Caemmerer S . 2017. Effects of reduced carbonic anhydrase activity on CO_2_ assimilation rates in *Setaria viridis*: a transgenic analysis. Journal of Experimental Botany 68: 299–310. 27702996 10.1093/jxb/erw357 PMC5853810

[nph18427-bib-0047] Parkhurst DF . 1994. Diffusion of CO_2_ and other gases inside leaves. New Phytologist 126: 449–479. 33874469 10.1111/j.1469-8137.1994.tb04244.x

[nph18427-bib-0048] Pathare VS , Koteyeva N , Cousins AB . 2020a. Increased adaxial stomatal density is associated with greater mesophyll surface area exposed to intercellular air spaces and mesophyll conductance in diverse C_4_ grasses. New Phytologist 225: 169–182. 31400232 10.1111/nph.16106

[nph18427-bib-0049] Pathare VS , Sonawane BV , Koteyeva N , Cousins AB . 2020b. C_4_ grasses adapted to low precipitation habitats show traits related to greater mesophyll conductance and lower leaf hydraulic conductance. Plant, Cell & Environment 43: 1897–1910. 10.1111/pce.13807 32449181

[nph18427-bib-0050] Peguero‐Pina JJ , Sancho‐Knapik D , Flexas J , Galmes J , Niinemets U , Gil‐Pelegrin E . 2016. Light acclimation of photosynthesis in two closely related firs (*Abies pinsapo* Boiss. and *Abies alba* Mill.): the role of leaf anatomy and mesophyll conductance to CO_2_ . Tree Physiology 36: 300–310. 26543153 10.1093/treephys/tpv114 PMC4885940

[nph18427-bib-0051] Pengelly JJ , Sirault XR , Tazoe Y , Evans JR , Furbank RT , von Caemmerer S . 2010. Growth of the C_4_ dicot *Flaveria bidentis*: photosynthetic acclimation to low light through shifts in leaf anatomy and biochemistry. Journal of Experimental Botany 61: 4109–4122. 20693408 10.1093/jxb/erq226 PMC2935879

[nph18427-bib-0052] Pfeffer M , Peisker M . 1998. CO_2_ gas exchange and phosphoenolpyruvate carboxylase activity in leaves of *Zea mays* L. Photosynthesis Research 58: 281–291.

[nph18427-bib-0053] Pons TL , Flexas J , von Caemmerer S , Evans JR , Genty B , Ribas‐Carbo M , Brugnoli E . 2009. Estimating mesophyll conductance to CO_2_: methodology, potential errors, and recommendations. Journal of Experimental Botany 60: 2217–2234. 19357431 10.1093/jxb/erp081

[nph18427-bib-0054] R Core Team . 2018. R: a language and environment for statistical computing. Vienna, Austria: R Foundation for Statistical Computing.

[nph18427-bib-0055] Rogers A , Medlyn BE , Dukes JS , Bonan G , von Caemmerer S , Dietze MC , Kattge J , Leakey ADB , Mercado LM , Niinemets Ü *et al*. 2017. A roadmap for improving the representation of photosynthesis in Earth system models. New Phytologist 213: 22–42. 27891647 10.1111/nph.14283

[nph18427-bib-0056] Sharwood RE , Ghannoum O , Kapralov MV , Gunn LH , Whitney SM . 2016. Temperature responses of Rubisco from Paniceae grasses provide opportunities for improving C_3_ photosynthesis. Nature Plants 2: 16186. 27892943 10.1038/nplants.2016.186

[nph18427-bib-0057] Shrestha A , Buckley TN , Lockhart EL , Barbour MM . 2018. The response of mesophyll conductance to short‐ and long‐term environmental conditions in chickpea genotypes. AoB Plants 11: ply073. 30680087 10.1093/aobpla/ply073 PMC6340285

[nph18427-bib-0058] Sonawane BV , Cousins AB . 2019. Uncertainties and limitations of using carbon‐13 and oxygen‐18 leaf isotope exchange to estimate the temperature response of mesophyll CO_2_ conductance in C_3_ plants. New Phytologist 222: 122–131. 30394538 10.1111/nph.15585

[nph18427-bib-0059] Sonawane BV , Cousins AB . 2020. Mesophyll CO_2_ conductance and leakiness are not responsive to short‐ and long‐term soil water limitations in the C_4_ plant *Sorghum bicolor* . The Plant Journal 103: 1590–1602. 32438487 10.1111/tpj.14849

[nph18427-bib-0060] Sonawane BV , Koteyeva NK , Johnson DM , Cousins AB . 2021. Differences in leaf anatomy determines temperature response of leaf hydraulic and mesophyll CO_2_ conductance in phylogenetically related C_4_ and C_3_ grass species. New Phytologist 230: 1802–1814. 33605441 10.1111/nph.17287

[nph18427-bib-0061] Studer AJ , Gandin A , Kolbe AR , Wang L , Cousins AB , Brutnell TP . 2014. A limited role for carbonic anhydrase in C_4_ photosynthesis as revealed by a ca1ca2 double mutant in maize. Plant Physiology 165: 608–617. 24706552 10.1104/pp.114.237602 PMC4044840

[nph18427-bib-0062] Tazoe Y , von Caemmerer S , Badger MR , Evans JR . 2009. Light and CO_2_ do not affect the mesophyll conductance to CO_2_ diffusion in wheat leaves. Journal of Experimental Botany 60: 2291–2301. 19255060 10.1093/jxb/erp035

[nph18427-bib-0063] Terashima I , Hanba YT , Tazoe Y , Vyas P , Yano S . 2006. Irradiance and phenotype: comparative eco‐development of sun and shade leaves in relation to photosynthetic CO_2_ diffusion. Journal of Experimental Botany 57: 343–354. 16356943 10.1093/jxb/erj014

[nph18427-bib-0064] Terashima I , Hanba YT , Tholen D , Niinemets Ü . 2011. Leaf functional anatomy in relation to photosynthesis. Plant Physiology 155: 108–116. 21075960 10.1104/pp.110.165472 PMC3075775

[nph18427-bib-0065] Tholen D , Boom C , Noguchi K , Ueda S , Katase T , Terashima I . 2008. The chloroplast avoidance response decreases internal conductance to CO_2_ diffusion in *Arabidopsis thaliana* leaves. Plant, Cell & Environment 31: 1688–1700. 10.1111/j.1365-3040.2008.01875.x 18721264

[nph18427-bib-0066] Tholen D , Zhu X‐G . 2011. The mechanistic basis of internal conductance: a theoretical analysis of mesophyll cell photosynthesis and CO_2_ diffusion. Plant Physiology 156: 90–105. 21441385 10.1104/pp.111.172346 PMC3091052

[nph18427-bib-0067] Tosens T , Niinemets Ü , Westoby M , Wright IJ . 2012. Anatomical basis of variation in mesophyll resistance in eastern Australian sclerophylls: news of a long and winding path. Journal of Experimental Botany 63: 5105–5119. 22888123 10.1093/jxb/ers171 PMC3430992

[nph18427-bib-0068] Ubierna N , Cernusak LA , Holloway‐Phillips M , Busch FA , Cousins AB , Farquhar GD . 2019. Critical review: incorporating the arrangement of mitochondria and chloroplasts into models of photosynthesis and carbon isotope discrimination. Photosynthesis Research 141: 5–31. 30955143 10.1007/s11120-019-00635-8

[nph18427-bib-0069] Ubierna N , Gandin A , Boyd RA , Cousins AB . 2017. Temperature response of mesophyll conductance in three C_4_ species calculated with two methods: ^18^O discrimination and *in vitro V* _pmax_ . New Phytologist 214: 66–80. 27918624 10.1111/nph.14359

[nph18427-bib-0070] Ubierna N , Gandin A , Cousins AB . 2018. The response of mesophyll conductance to short‐term variation in CO_2_ in the C_4_ plants *Setaria viridis* and *Zea mays* . Journal of Experimental Botany 69: 1159–1170. 29474683 10.1093/jxb/erx464 PMC6018935

[nph18427-bib-0071] Uehlein N , Otto B , Hanson DT , Fischer M , McDowell N , Kaldenhoff R . 2008. Function of Nicotiana tabacum aquaporins as chloroplast gas pores challenges the concept of membrane CO_2_ permeability. Plant Cell 20: 648–657. 18349152 10.1105/tpc.107.054023 PMC2329941

[nph18427-bib-0072] Veromann‐Jürgenson L‐L , Tosens T , Laanisto L , Niinemets Ü . 2017. Extremely thick cell walls and low mesophyll conductance: welcome to the world of ancient living! Journal of Experimental Botany 68: 1639–1653. 28419340 10.1093/jxb/erx045 PMC5441924

[nph18427-bib-0073] Wright IJ , Reich PB , Westoby M , Ackerly DD , Baruch Z , Bongers F , Cavender‐Bares J , Chapin T , Cornelissen JHC , Diemer M *et al*. 2004. The worldwide leaf economics spectrum. Nature 428: 821–827. 15103368 10.1038/nature02403

[nph18427-bib-0074] Xiong D , Flexas J . 2021. Leaf anatomical characteristics are less important than leaf chemical properties in determining photosynthesis responses to top‐dress N. Journal of Experimental Botany 72: 5709–5720. 34022050 10.1093/jxb/erab230

[nph18427-bib-0075] Xiong D , Liu X , Liu L , Douthe C , Li Y , Peng S , Huang J . 2015. Rapid responses of mesophyll conductance to changes of CO_2_ concentration, temperature and irradiance are affected by N supplements in rice. Plant, Cell & Environment 38: 2541–2550. 10.1111/pce.12558 25923314

[nph18427-bib-0076] Yin X , Struik PC . 2009. Theoretical reconsiderations when estimating the mesophyll conductance to CO_2_ diffusion in leaves of C_3_ plants by analysis of combined gas exchange and chlorophyll fluorescence measurements. Plant, Cell & Environment 32: 1513–1524. 10.1111/j.1365-3040.2009.02016.x 19558403

[nph18427-bib-0077] Zar JH . 2007. Biostatistical analysis, *5^th^ edn* . Upper Saddle River, NJ, USA: Prentice-Hall.

