# Supplementary material for: Mesophyll conductance response to short‐term changes in p CO2 is related to leaf anatomy and biochemistry in diverse C4 grasses
Source: New Phytol. 2022 Sep 1;236(4):1281–95. doi: 10.1111/nph.18427 (PMC9825963; doi:10.1111/nph.18427)
Supplement: Supplementary file 1 — Fig. S1 Sensitivity of mesophyll conductance (g m; estimated by Ogee et al., 2018 method) to changes in fraction of CO2 not produced in bundle sheath cells (ϕr). Fig. S2 Sensitivity of mesophyll conductance (g m; estimated by Ogee et al., 2018 method) to changes in leaf temperature (T leaf). Fig. S3 Relationship of model coefficient a (indicating value of g m at 34 Pa pCO2) with coefficient b (sensitivity of g m to C a; relatively lower b values indicate lower rate of change in g m with C a) and relationship between coefficient b and percentage change in g m. Fig. S4 Relationship of percent increase in g m with mesophyll cell wall thickness (T CW) mesophyll surface area exposed to intercellular air spaces (Smes) ratio of T CW : Smes, stomatal ratio (SR), stomatal density adaxial (SDada) and leaf thickness among the 16 C4 grasses measured in current study. Fig. S5 Relationship of percent increase in g m with PEPC activity Rubisco activity CA activity expressed as k CA, PEPC affinity for HCO3 − (K m), maximum photosynthetic capacity (A max) and leaf N content (Narea) among the 16 C4 grasses measured in current study. Fig. S6 Relationship of percent increase in g m with ratio of mesophyll cell wall thickness (T CW) to PEPC activity Rubisco activity PEPC affinity for HCO3 − (K m), CA activity expressed as k CA, maximum photosynthetic rates (A max) and leaf N content (Narea) among the 16 C4 grasses measured in current study. Fig. S7 Relationship of percent increase in g m with percent decrease in leaf‐level water‐use efficiency (TEi) expressed as A net/g sw (higher negative value indicates greater decrease in TEi), percent increase in stomatal conductance to water (g sw) and percent decrease in net photosynthetic rates (A net) (higher negative value indicates greater decrease in A net) for the 16 C4 grasses measured in current study. Fig. S8 PCA biplot showing major axes of variation in important leaf‐level anatomical and biochemical traits and percent change (increase o [file NPH-236-1281-s001.pdf]

New Phytologist

New Phytologist

Supporting Information

Article title: **Mesophyll conductance response to short-term changes in *p*CO_2 is related to leaf anatomy and biochemistry in diverse C_4 grasses**

Authors: Varsha S. Pathare[1]*, Robert J. DiMario[1], Nuria Koteyeva[1,2] and Asaph B. Cousins[1]
1. School of Biological Sciences, Washington State University, Pullman, Washington, USA 99164-4236. 2. Laboratory of Anatomy and Morphology, V.L. Komarov Botanical Institute of the Russian Academy of Sciences, 197376 St. Petersburg, Russia

'Article acceptance date: 31 July 2022'

The following Supporting Information is available for this article:

Methods S1 ***Measurement of anatomical traits:*** Light microscopy images of leaf cross sections were used to measure length of mesophyll cell walls exposed to intercellular air spaces (IAS) using 10-15 different fields of view for each leaf (*n* = 3 per species) taken at x 50 and x 100 magnifications. The S_{mes} was calculated from measurements of total length of mesophyll cell walls exposed to IAS and width of section analyzed using equation from Evans *et al.*, (1994) with curvature correction factor (F) of 1.34. T_{CW} was measured from TEM micrographs using at least 15 images for each leaf. Values for all the leaf anatomical traits used in current study have been published (Pathare *et al*., 2020).

Methods S2 ***Principal Component Analysis:*** To complement the regression analysis, we also performed a PCA (R package FactoMineR; Le *et al.*, 2008) using the important leaf-level anatomical and biochemical traits and percent changes in g_m, A_{net}, g_{sw} and TE_i and mean annual precipitation. The PCA was performed on leaf traits from 16 C_4 grasses, where the first four axes with eigenvalues ≥ 1 were retained for analysis (Table S4). The first two major axes (PC1 and PC2) of leaf trait variation along with the average position of C_4 grasses in PC1-PC2 space are presented in Figure S8. All traits were scaled during the analysis. The first four principal components (PC) had eigenvalues greater than one (Table S4) and were retained according to Kaiser's rule (Kaiser, 1960). For each trait, factor loadings greater than 0.5 in absolute value were considered important. The first major PC1 explains about 44 % of total variation in the C_4 grasses and has a positive association with key anatomical and biochemical traits and percent change in g_m and TE_i. Whereas PC1 showed negative association with T_{CW} and percent change in g_{sw} and mean annual precipitation. Thus, PC1 delineates the C_4 grasses into those with greater CO_2 response of g_m (higher PC1; greater enzyme activity and lower T_{CW}) and those with lower CO_2 response of g_m (lower PC1; lower enzyme activity and greater T_{CW}). MAP also was strongly related to PC1, with species from drier habitats showing greater CO_2 response of g_m. Alternatively, PC2 explains about 18 % of the total variation in the C_4 grasses and is positively associated with K_m and percent change in A_{net} and TE_i and negatively associated with T_{CW} and k_{CA}. PC2 tends to separate the C_4 grasses into those showing greater K_m values, lower T_{CW} and lesser decrease in A_{net} and TE_i at low pCO_2.

Table S1 Key input parameters used in calculation of isotope parameters and estimation of mesophyll conductance (g_m) for the 16 C_4 grasses at four pCO_2 levels (34, 27, 20 and 14 Pa) and a temperature of 25 ^oC using the Ogee *et al.*, 2018 method. Further details of equations and calculations of fractionation factors can be found in original publications (Ubierna *et al.*, 2017; Ogee *et al.*, 2018).

**Parameter (units)**	**Description**	**Value used in current study**
A_{net} (µmol $m^{-2}s^{-1}$)	Net photosynthetic rates	Measured for each species
E (mol $m^{-2}s^{-1}$)	Transpiration	Measured for each species
Atmospheric pressure in chamber (kPa)		91 kPa
K_{CA} (µmol m^{-2} s^{-1} Pa^{-1})	Activity of carbonic anhydrase expressed as first order rate constant	Measured for each species
C_a (Pa)	CO_2 partial pressure in ambient air	Measured for each species
C_i (Pa)	CO_2 partial pressure inside the leaf	Measured for each species
Cs (or C_L; Pa)	CO_2 partial pressure at the leaf surface	Calculated for each species
C_m (Pa)	CO_2 partial pressure in the mesophyll cytosol at the sites of carbonic anhydrase (CA)	Calculated for each species using the Ogee *et al.*, 2018 method (Ogee *et al.*, 2018)
ρi (unitless)	$A_{net}/K_{CA}*C_i$	Calculated for each species
$S_{C/O}$ (Pa Pa^{-1})	Rubisco CO_2/O_2 specificity	1310 at 25°C (Boyd *et al.*, 2015)
γ*	Half of the reciprocal of Rubisco specificity	Calculated as $\gamma* = 0.5/S_{C/O}$
Γ* (Pa)	Compensation point in the absence of mitochondrial respiration	Used a global value of 6 (Pa Pa^{-1}) for all C_4 species (Sharwood *et al.*, 2016)
Vr (µmol $m^{-2}s^{-1}$)	Non-photorespiratory CO_2 released during the day or day respiration	Measured dark respiration (R_n) for each species and assumed $Vr = R_n$
ϕr (unitless)	Fraction of respired CO_2 not produced in the bundle sheath cells of C_4 plants	Assumed to be 0.5 (von Caemmerer, 2000)
δ^{18}_{in} and δ^{18}_{a} (‰ vSMOW)	$\delta^{18}O$ of the CO_2 in the air entering (δ^{18}_{in}) and leaving (δ^{18}_{a}) the leaf chamber	Measured for each species
δ^{18}_{w-in} and δ^{18}_{w-a} (‰ vSMOW)	$\delta^{18}O$ of the water vapour in the air entering (δ^{18}_{w-in}) and leaving (δ^{18}_{w-a}) the leaf chamber	Measured for each species
w_{in} and w_a (mmol mol^{-1})	Water vapor mole fraction in the air entering (w_{in}) and leaving (w_a) the leaf chamber	Measured for each species
g_{bl} (mol m^{-2} s^{-1})	Boundary layer conductance	Measured for each species
g_{sw} (mol m^{-2} s^{-1})	Stomatal conductance to water vapour	Measured for each species
g_{ac} (mol m^{-2} s^{-1})	Stomatal conductance to CO_2	Measured for each species
pH	Mesophyll cell pH	Assumed to be 7.2
T_{leaf}	Leaf temperature	25°C

Table S2 Results of one-way ANOVA with species as main effects for all the leaf-level anatomical and biochemical traits measured for 16 C_4 grasses in current study.

Traits measured	Degrees of freedom	*F*-value	*P*-value
T_{CW}	15	12.14	**< 0.001**
S_{mes}	15	11.37	**< 0.001**
SR	15	88.24	**< 0.001**
SD_{ada}	15	60.16	**< 0.001**
Leaf thickness	15	14.94	**< 0.001**
PEPC activity	15	39.4	**< 0.001**
Rubisco activity	15	31.72	**< 0.001**
k_{CA}	15	71.6	**< 0.001**
K_m	15	16.94	**< 0.001**
A_{max}	15	9.5	**< 0.001**
N_{area}	15	10.14	**< 0.001**

Statistically significant *P*-values (≤ 0.05) are highlighted in bold. Numerator degrees of freedom and *F*-values (*F* test statistic) are also shown. Mesophyll cell wall thickness (T_{CW}), total mesophyll cell surface area exposed to intercellular air space per unit of leaf surface area (S_{mes}), stomatal ratio (SR), adaxial stomatal density (SD_{ada}), phosphoenolpyruvate carboxylase activity (PEPC), Carbonic anhydrase activity expressed as first-order rate constant (k_{CA}), PEPC's affinity for HCO_3^- (K_m), maximum photosynthetic capacity (A_{max}) and leaf N content (N_{area}).

Table S3 C_4 grasses used in current study along with their biochemical subtype and mean ± 1 SE values for the equation ($g_m = a \times (34/C_a)^b$) constants (a and b) derived for the CO_2 response of g_m in these 16 C_4 grasses. Average values of a and b for three C_4 biochemical subtypes are also shown. Corresponding letters of post-hoc Tukey's test are also shown. Coefficient a indicates the value of g_m at 34 Pa pCO_2, whereas coefficient b indicates the rate of change in g_m with decrease in pCO_2. Responses of g_m to changes in pCO_2 inside leaf chamber (C_a) along with the model line are shown in Fig.1. The species are identified by the assigned species code in the graphs.

Species	Species code	Biochemical subtype	Coefficient a	Coefficient b
Chloris gayana Kunth.	cgay	PCK	8.03 ± 0.23 bcde	0.93 ± 0.09 abc
Danthoniopsis dinteri (Pilg.) C.E.Hubb.	ddin	NADP-ME	16.32 ± 1.83 a	0.82 ± 0.18 abc
Digitaria sanguinalis (L.) Scop.	dsan	NADP-ME	6.45 ± 0.59 bcd	0.44 ± 0.02 ab
Eriachne aristidea E. Muell.	eari	NADP-ME	10.18 ± 0.11 be	1.29 ± 0.15 c
Echinochloa colona (L.) Link.	ecol	NADP-ME	6.96 ± 0.28 bcde	0.58 ± 0.13 ab
Eragrostis curvula (Schrad.) Nees.	ecur	NAD-ME	7.15 ± 0.34 bcde	0.38 ± 0.08 b
Eleusine indica (L.) Gaertn.	eind	NAD-ME	10.46 ± 1.49 e	0.34 ± 0.15 b
Eriochloa sericea Munro ex Vasey.	eser	PCK	5.53 ± 0.41 cd	0.87 ± 0.08 abc
Heteropogon contortus Beauv.	hcon	PCK	7.02 ± 0.86 bcde	0.21 ± 0.07 b
Ischaemum afrum (J.F.Gmel.) Dandy.	iafr	NADP-ME	9.37 ± 0.78 bce	0.52 ± 0.13 ab
Paspalum dilatatum Poir.	pdil	NADP-ME	4.72 ± 0.13 d	0.4 ± 0.07 b
Paspalum macrophyllum Kunth	pmac	NADP-ME	6.42 ± 0.22 bcd	0.28 ± 0.06 b
Panicum maximum Jacq.	pmax	PCK	7.8 ± 0.65 bcde	0.51 ± 0.12 ab
Panicum miliaceum L.	pmil	NAD-ME	8.99 ± 1.15 bce	0.72 ± 0.17 abc
Panicum virgatum L.	pvir	NAD-ME	8.49 ± 0.2 bcde	0.62 ± 0.08 abc
Urochloa dictyoneura (Fig. & De Not.) Veldkamp.	udic	PCK	6.69 ± 0.35 bcde	1.13 ± 0.32 ac

**Mean ± 1 SE values for coefficients a and b for the three biochemical subtypes of C_4 species**		**Coefficient a**	**Coefficient b**
	NAD-ME	8.77 ± 0.68 a	0.512 ± 0.09 a
	NADP-ME	8.63 ± 1.46 a	0.618 ± 0.13 a
	PCK	7.01 ± 0.44 a	0.73 ± 0.16 a

Table S4 Component loadings for important leaf-level traits determined on 16 C_4 grasses.

**Eigenvalues**	7.03	2.88	1.84	1.25
**Total variance explained (%)**	44	18	11.5	7.8
**Traits**	**PC1**	**PC2**	**PC3**	**PC4**
% change in g_{sw}	**-0.63**	0.26	**0.72**	-0.07
MAP	**-0.6**	-0.09	-0.13	0.36
T_{CW}	**-0.55**	**-0.7**	-0.03	-0.08
% change in A_{net}	-0.05	**0.88**	0.4	0
K_m	-0.03	**0.65**	-0.43	-0.41
% change in TE_i	**0.57**	**0.63**	-0.42	0.15
% change in g_m	**0.63**	0.36	-0.45	-0.02
k_{CA}	**0.65**	-0.48	-0.35	0.18
SD_{ada}	**0.68**	0.24	0.41	0.3
PEPC activity	**0.7**	-0.09	0.14	**-0.61**
A_{max}	**0.74**	0.22	0.27	0.3
N_{area}	**0.74**	-0.35	0.24	0.19
S_{mes}	**0.77**	-0.23	0.36	-0.3
SR	**0.84**	-0.08	0.02	0.29
Rubisco activity	**0.85**	-0.26	0.08	-0.35
Leaf thickness	**0.87**	-0.02	0.02	0.1

Eigenvalues and total variance explained (%) by first four principal components (PC1, PC2, PC3, PC4) is shown. The first four PCs have eigenvalues ≥ 1 and hence are retained. Component loadings greater than 0.5 in absolute value are shown in bold face. Mean annual precipitation (MAP), Mesophyll cell wall thickness (T_{CW}), intrinsic water use efficiency (TE_i), stomatal

conductance to water vapor diffusion (g_{sw}), net photosynthetic rate per unit of leaf surface area (A_{net}), total mesophyll cell surface area exposed to intercellular air space per unit of leaf surface area (S_{mes}), mesophyll conductance to CO_2 diffusion estimated by Ogee *et al.*, 2018 method (g_m), stomatal ratio (SR), adaxial stomatal density (SD_{ada}), Carbonic anhydrase activity expressed as first-order rate constant (k_{CA}), phosphoenolpyruvate carboxylase activity (PEPC), PEPC's affinity for HCO^-_3 (K_m), maximum photosynthetic capacity (A_{max}) and leaf N content on area basis (N_{area}).

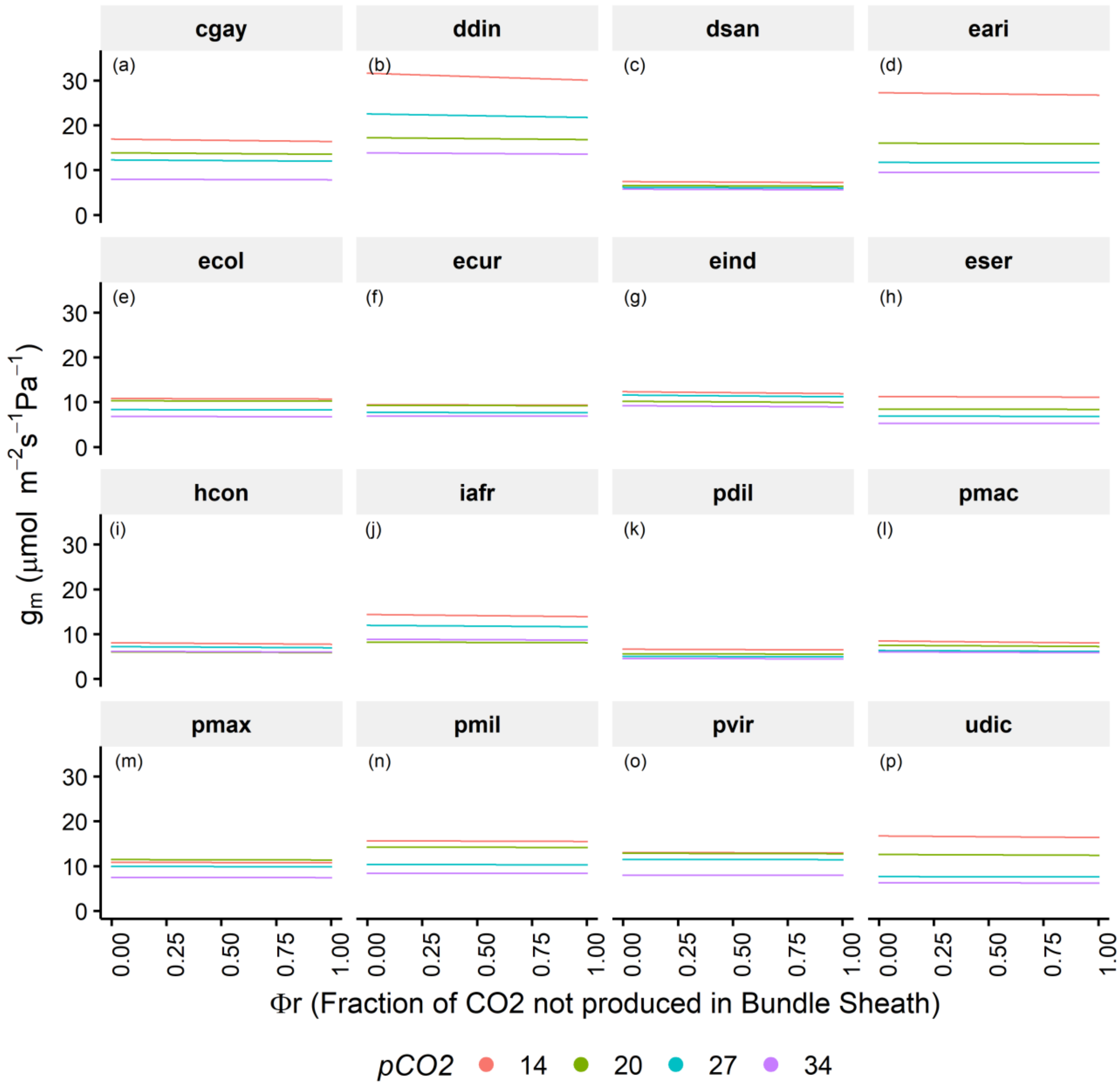

Fig. S1 Sensitivity of mesophyll conductance (g_m; estimated by Ogee *et al*., 2018 method) to changes in fraction of CO_2 not produced in Bundle Sheath cells (ϕr). Data for each of the species is shown separately from panel a to p. Sensitivity analysis was performed by calculating g_m using values of ϕr ranging from 0 to 1 (von Caemmerer, 2000) for each species and at all the four levels of pCO_2 inside chamber (C_a) used in current study (14, 20, 27, 34 Pa).

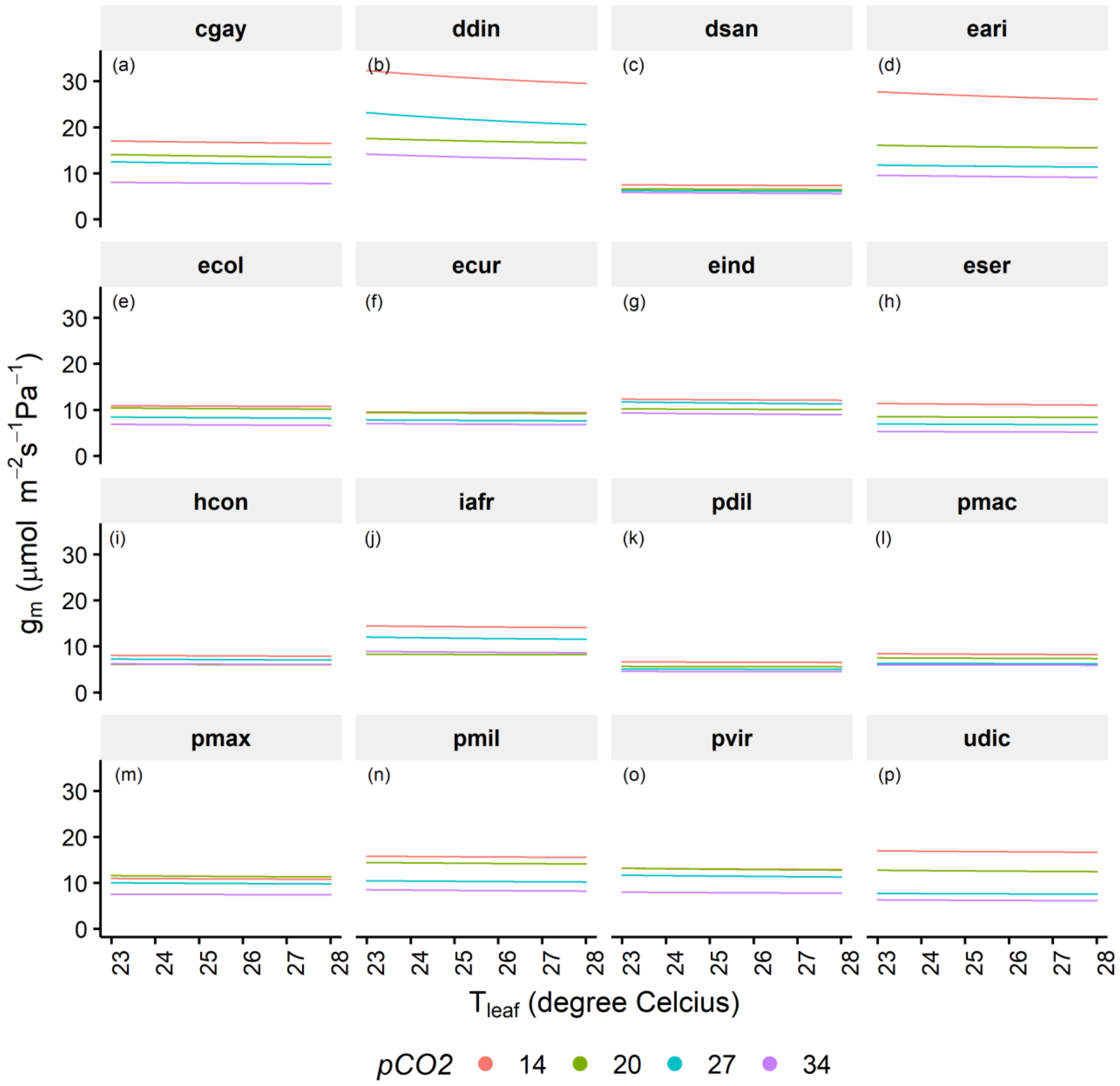

Fig. S2 Sensitivity of mesophyll conductance (g_m; estimated by Ogee *et al*., 2018 method) to changes in leaf temperature (T_{leaf}). Data for each of the species is shown separately from panel a to p. Sensitivity analysis was performed by calculating g_m using values of T_{leaf} values ranging from 23 to 28 degree Celsius for each species and at all the four levels of pCO_2 inside chamber (C_a) used in current study (14, 20, 27, 34 Pa).

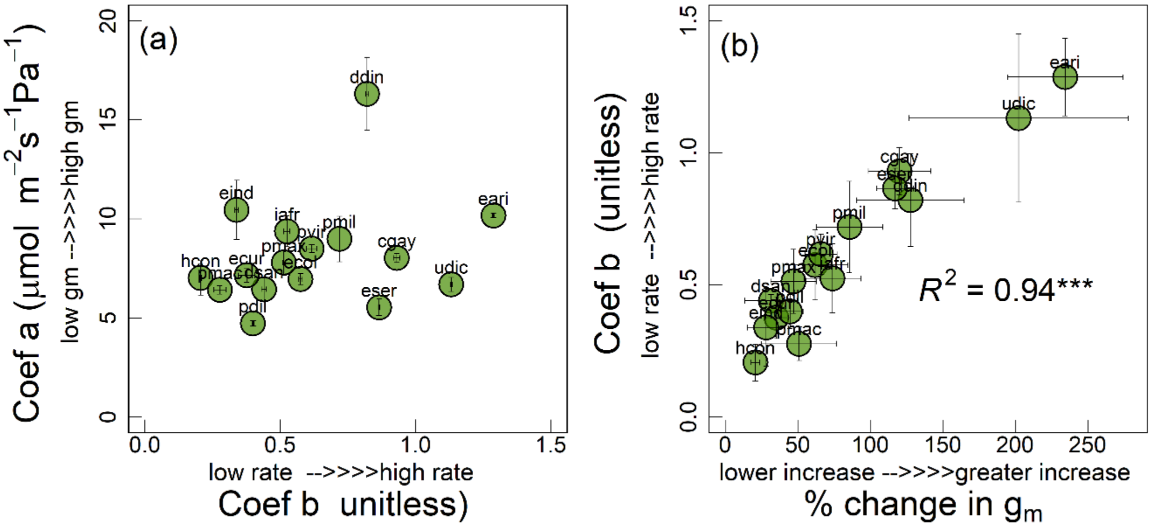

Fig. S3 Relationship of (a) model coefficient a (indicating value of g_m at 34 Pa pCO_2) with coefficient b (sensitivity of g_m to C_a; relatively lower b values indicate lower rate of change in g_m with C_a). Panel (b) shows relationship of ratio of coefficient b (used as proxy for CO_2 response of g_m) with percent change in g_m (calculated as: g_m at 14 Pa pCO_2- g_m at 34 Pa pCO_2) x 100/ (g_m at 34 Pa pCO_2)). Significance of regression coefficients (R^2): ***, $p \leq 0.001$. In panel (a), R^2 was derived after removing the outlier species ddin (with ddin, R^2 = 0.04). Each circle represents mean ± 1 SE value for each species (n = 3-6)

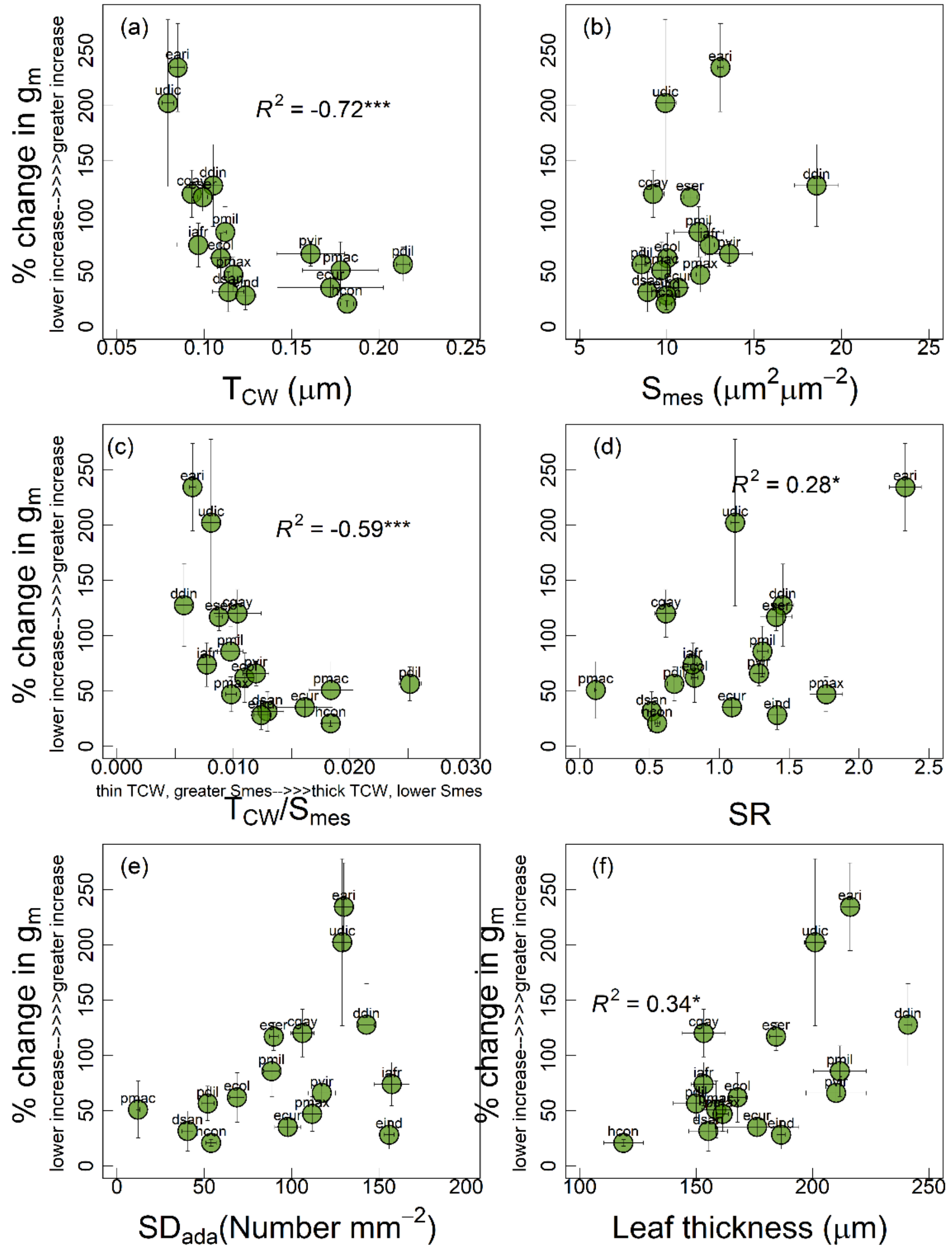

Fig. S4 Relationship of percent increase in g_m with (a) mesophyll cell wall thickness (T_{CW}) (b) mesophyll surface area exposed to intercellular air spaces (S_{mes}) (c) ratio of T_{CW}/S_{mes}, (d) stomatal ratio (SR), (e) stomatal density adaxial (SD_{ada}) and (f) leaf thickness among the 16 C_4 grasses measured in current study. Significance of regression coefficients (R^2): *, $p \leq 0.05$ and ***, $p \leq 0.001$. Each circle represents mean ± 1 SE value for each species (n = 3-6). Species names are indicated by codes given in Table 1.

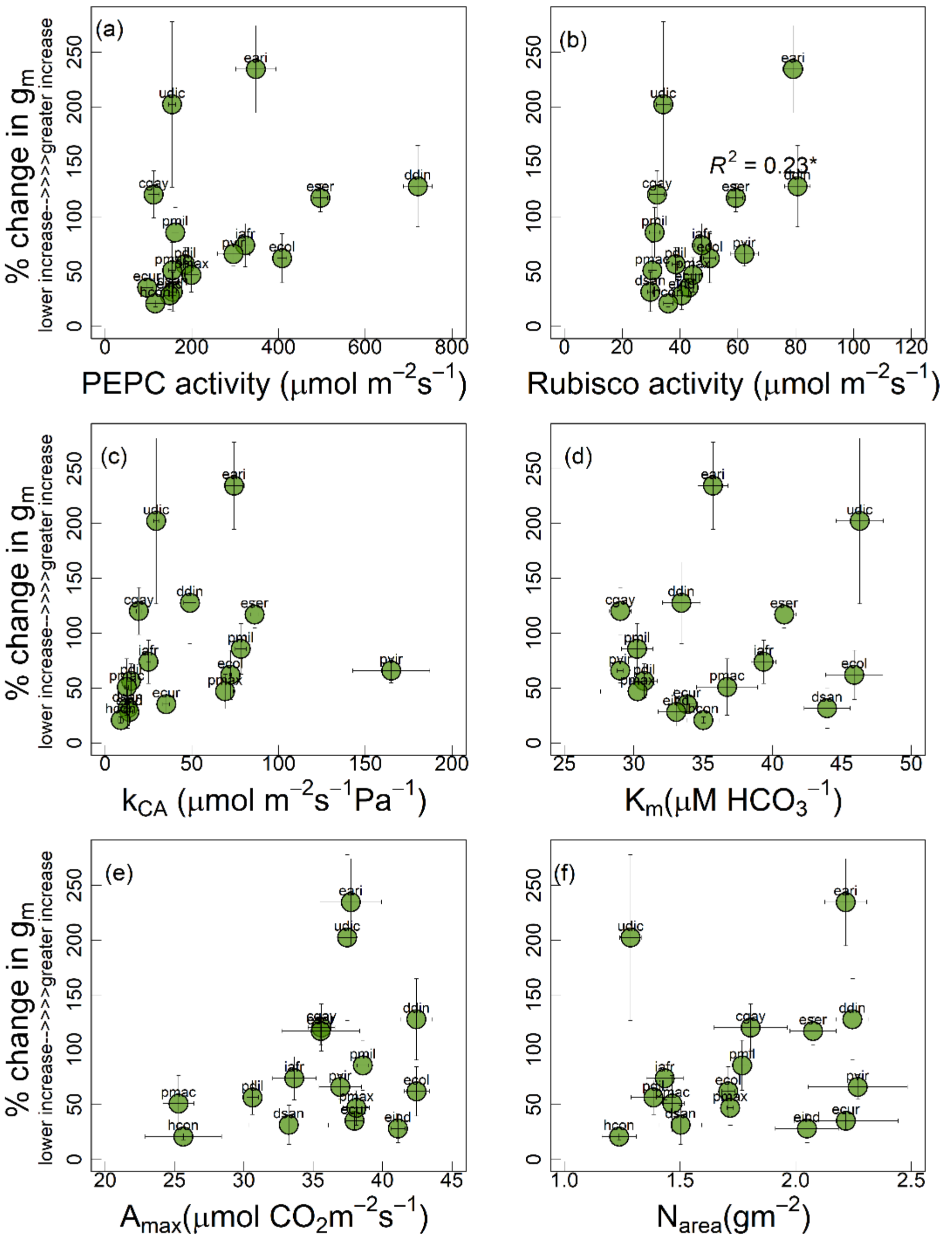

Fig. S5 Relationship of percent increase in g_m with (a) PEPC activity (b) Rubisco activity (c) CA activity expressed as k_{CA}, (d) PEPC's affinity for HCO_3^- (K_m), (e) maximum photosynthetic capacity (A_{max}) and (f) leaf N content (N_{area}) among the 16 C_4 grasses measured in current study. Significance of regression coefficients: *, $p \leq 0.05$. Linear models were used for deriving regression coefficients (R^2) in all panels. Each circle represents mean ± 1 SE value for each species (n = 3-6). Species names are indicated by codes given in Table 1. In panel (c), $R^2 = 0.04^{ns}$ after removing the influential species pvir.

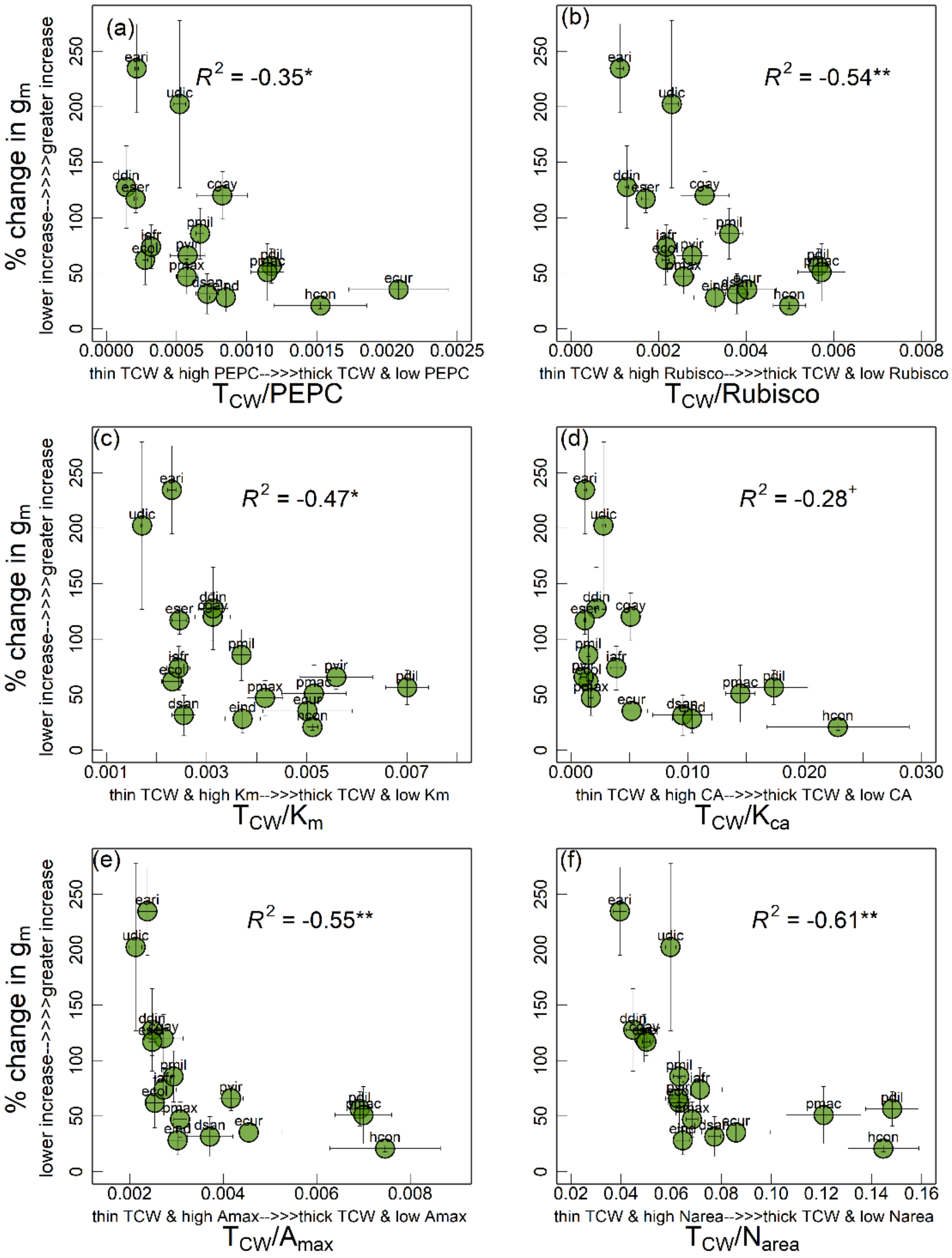

Fig. S6 Relationship of percent increase in g_m with ratio of mesophyll cell wall thickness (T_{CW}) to (a) PEPC activity (b) Rubisco activity (c) PEPC's affinity for HCO_3^- (K_m), (d) CA activity expressed as k_{CA}, (e) maximum photosynthetic rates (A_{max}) and (f) leaf N content (N_{area}) among the 16 C_4 grasses measured in current study. Significance regression coefficients (R^2): ^{ns}, non-significant, +, marginally significant, *, $p \leq 0.05$, **, $p \leq 0.01$ and ***, $p \leq 0.001$. Polynomial models were used for deriving regression coefficients (R^2) in all panels, except panel (a) and (d). Each circle represents mean ± 1 SE value for each species (n = 3-6). Species names are indicated by codes given in Table 1.

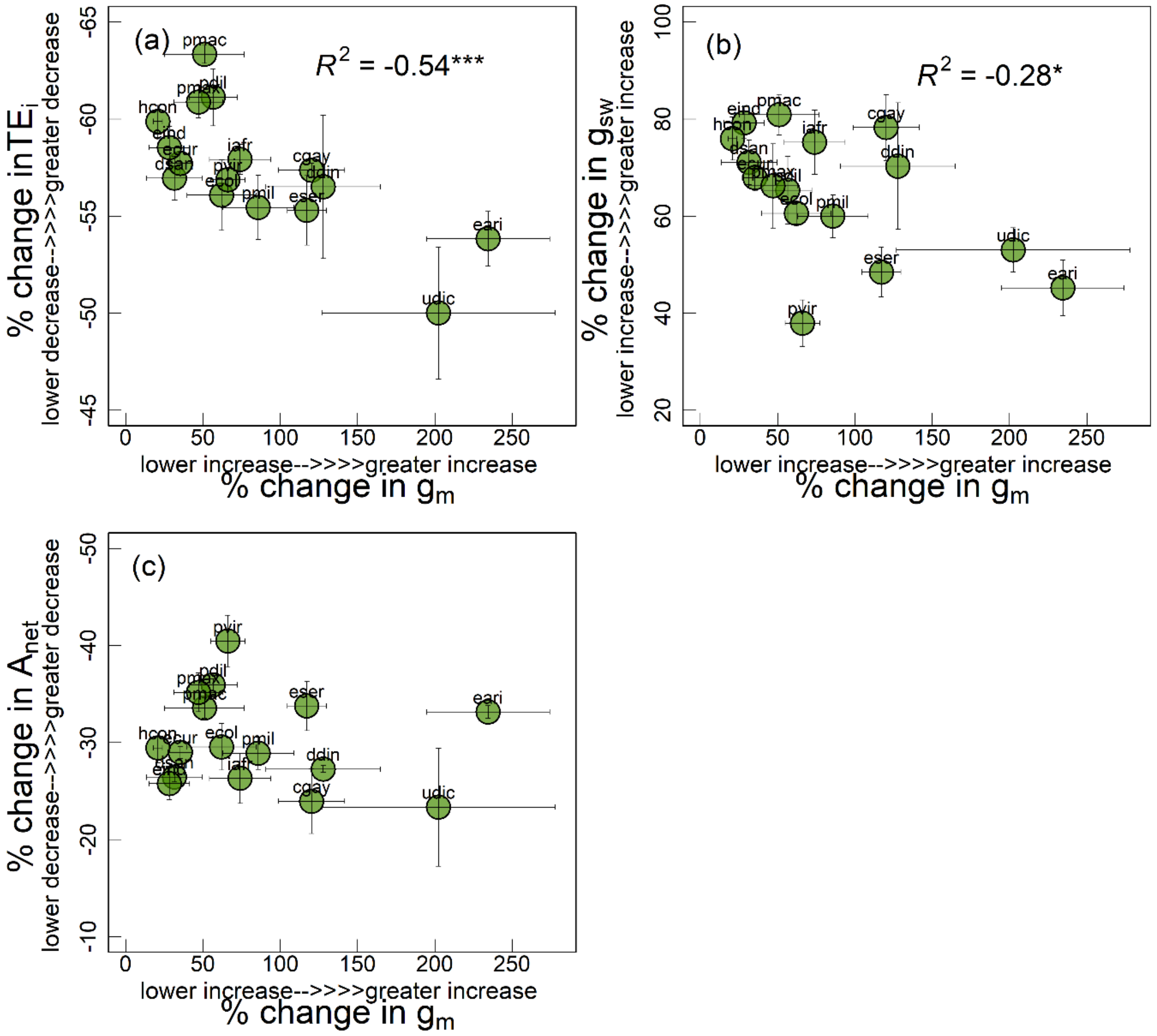

Fig. S7 Relationship of percent increase in g_m with (a) percent decrease in leaf-level water-use efficiency (TE_i) expressed as A_{net}/g_{sw} (higher negative value indicates greater decrease in TE_i), (b) percent increase in stomatal conductance to water (g_{sw}) and (c) percent decrease in net photosynthetic rates (A_{net}) (higher negative value indicates greater decrease in A_{net}) for the 16 C_4 grasses measured in current study. Each circle represents mean ± SE value for each species (n = 3-6). In panel (b), R^2 = -0.50** after removing the influential species pvir. Significance of regression coefficients (R^2): *, $p \leq 0.05$, **, $p \leq 0.01$ and ***, $p \leq 0.001$. Each circle represents mean ± 1 SE value for each species (n = 3-6). Species names are indicated by codes given in Table 1.

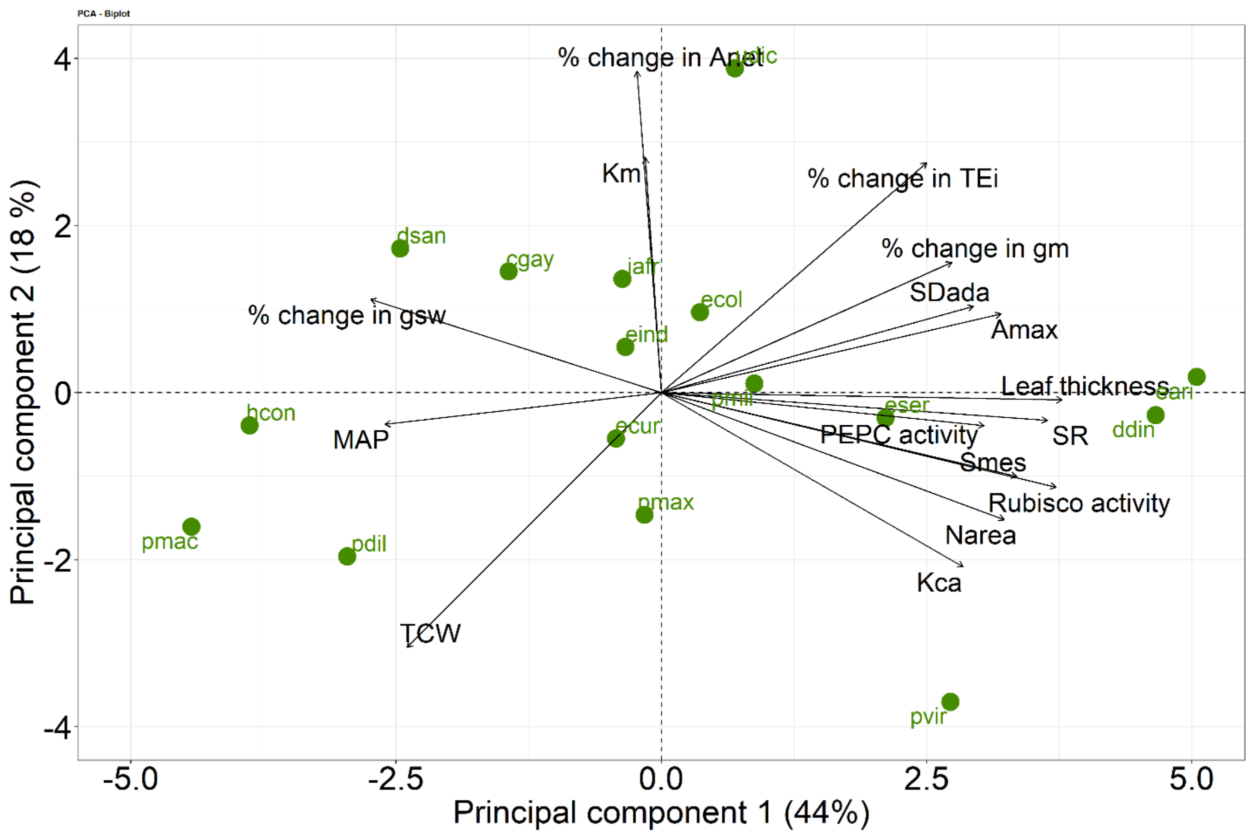

Fig. S8 PCA biplot showing major axes of variation in important leaf-level anatomical and biochemical traits and percent change (increase or decrease) in response to CO_2 in physiological traits like g_m, A_{net}, g_{sw} and TEi for the 16 diverse C_4 grasses measured in current study. Eigenvalues and factor loadings for first three principal components (PC) are shown in Table S1. The arrows are the vectors showing the correlation (across the C_4 grasses) between a trait and the PCs. Position of species in principal component space is shown in blue circles. Species names correspond to the description in Table 1. Values for MAP and anatomical traits were obtained from Pathare *et al.*, 2020a, b, whereas values for K_m were obtained from DiMario *et al.*, 2021. Points are mean values with n = 3-6 per species (mean ± SE values are given in Table 1). g_m, mesophyll conductance to CO_2 diffusion; SD_{ada}, adaxial stomatal density; S_{mes}, total mesophyll cell surface area exposed to intercellular air space per unit of leaf surface area; SR, stomatal ratio; N_{area}, leaf N content expressed on area basis; A_{max}, maximum photosynthetic rates at saturating light and pCO_2; PEPC, phosphoenolpyruvate carboxylase; Rubisco, ribulose-1,5-bisphosphate carboxylase/oxygenase; Kca, activity of Carbonic anhydrase expressed as first order rate constant; K_m, PEPC's affinity for HCO_3^-; A_{net}, net CO_2 assimilation rates; g_{sw}, stomatal conductance to water; TE_i, leaf-level water-use efficiency (A_{net}/g_{sw}); T_{CW}, mesophyll cell wall thickness; MAP, mean annual precipitation.

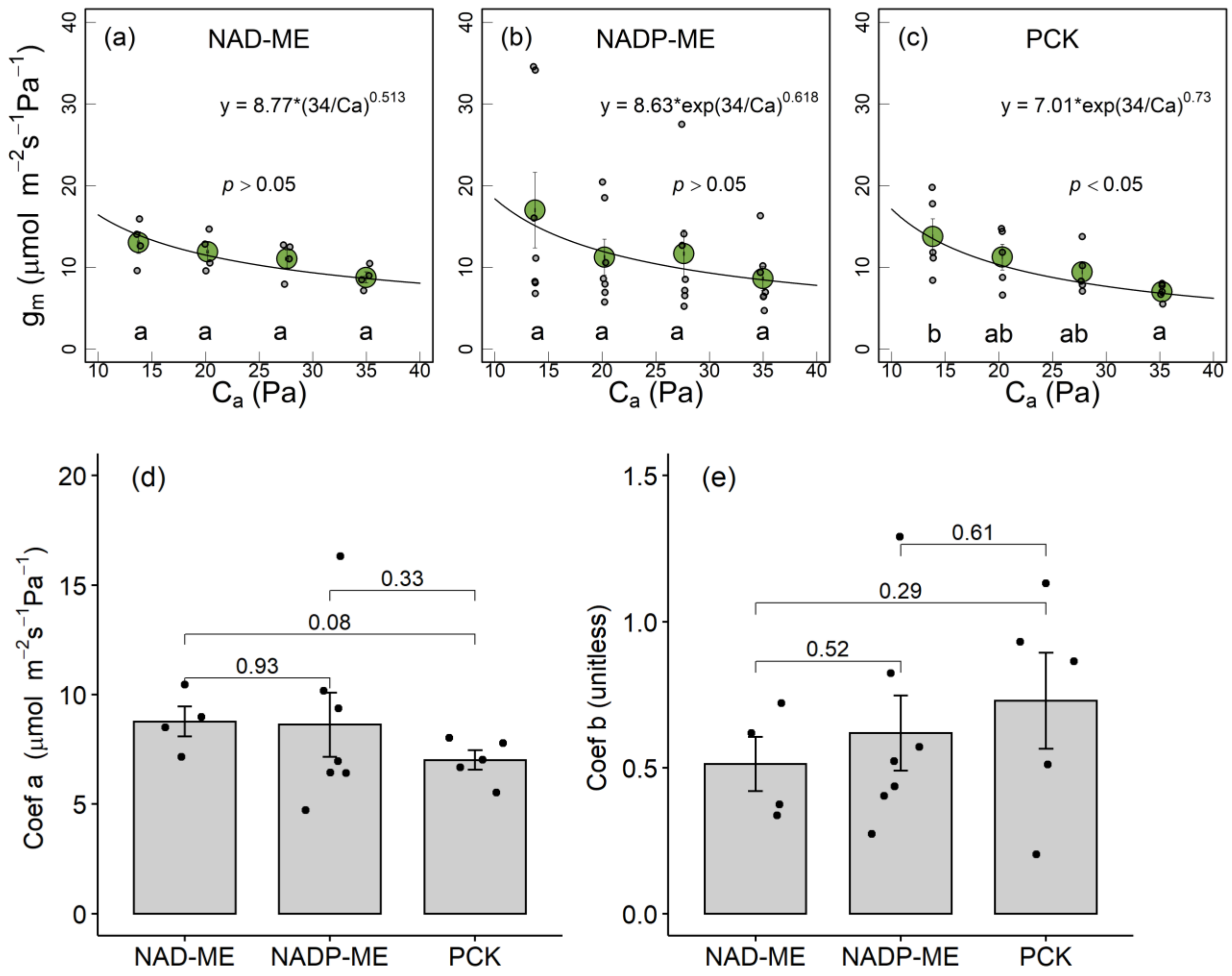

Fig. S9 Response of mesophyll conductance (g_m) to changes in *p*CO_2 inside leaf chamber (C_a) in three C_4 biochemical subtypes (NAD-ME, NADP-ME, PCK). Data for each of the subtypes is shown separately from panels a to c. CO_2 response of g_m (black solid line) modelled using equation $g_m = a \times (34/Ca)^b$ is shown, wherein coefficient a is the value of g_m at 34 Pa *p*CO_2 and b is the sensitivity of g_m to changes in C_a. Mean ± SE values for the model constants (a and b) for each subtype are shown in Table S3. Panel (d) and (e) shows the pair-wise comparisons (t-test *P*-values) of the coefficients among the subtypes. Measurements were performed at constant light (photosynthetic photon flux density (PPFD) = 1200 µmol m^{-2} s^{-1}) and leaf temperature (25°C). Values in each panel represent mean ± 1 SE (green color) and gray points indicate the replicate values for each subtype and CO_2 level (n = 4 for NAD-ME, n = 7 for NADP-ME and n = 5 for PCK). *P*-values from one-way ANOVA and Tukey's letters are shown.

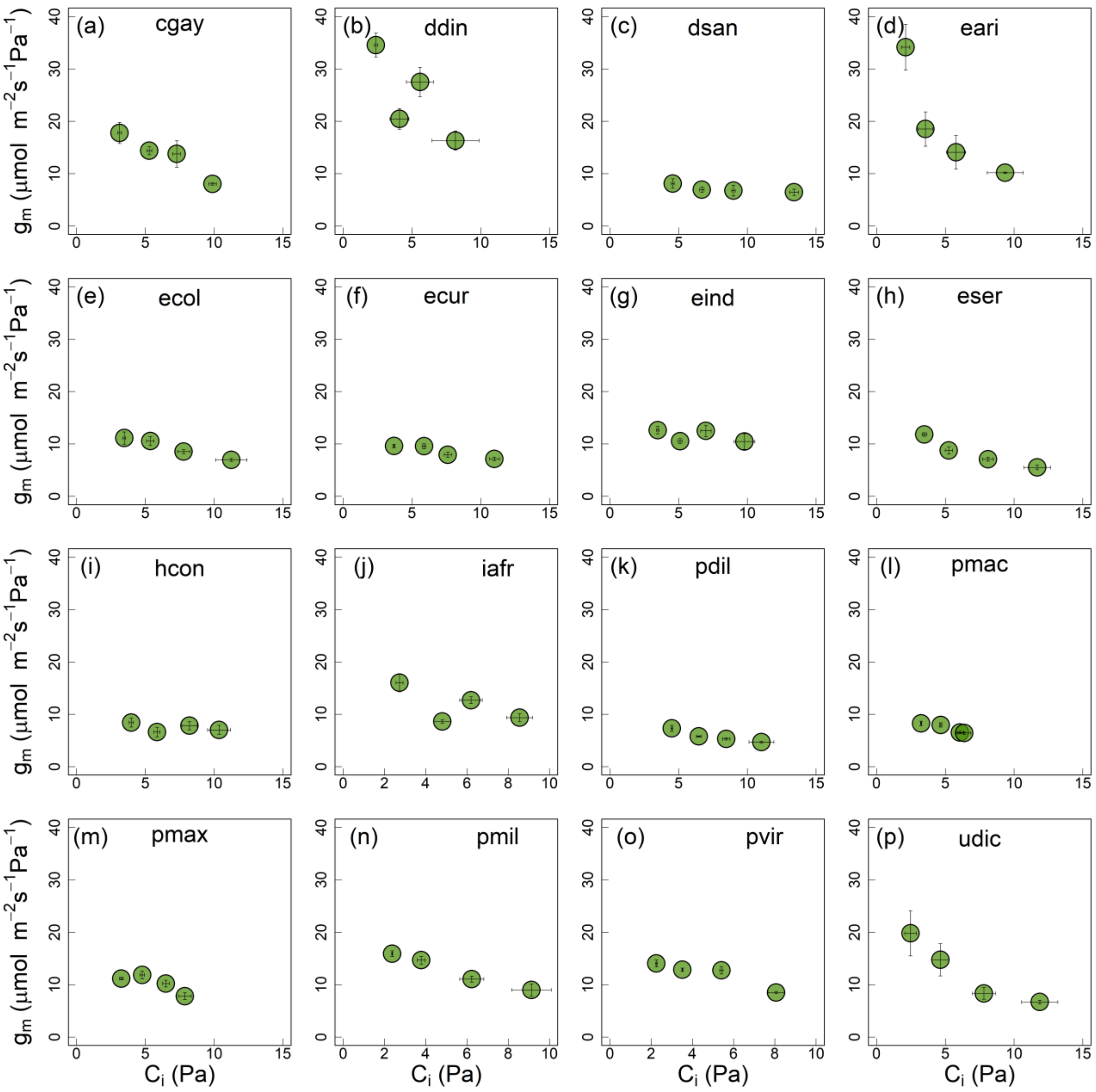

Fig. S10 Response of mesophyll conductance to CO_2 (g_m) to changes in intercellular CO_2 (C_i) in 16 diverse C_4 grasses measured in current study. Data for each of the species is shown separately from panel a to p. Measurements were performed at constant light (photosynthetic photon flux density (PPFD) = 1200 µmol m^{-2} s^{-1}) and leaf temperature (25°C). Values in each panel represent mean ± 1 SE (green color) with *n* = 3-6. Response of g_m to C_i for each species is plotted in separate panel. Species code has been indicated by first letter of genera and first three letters of species (see Table 1 for full names of species).

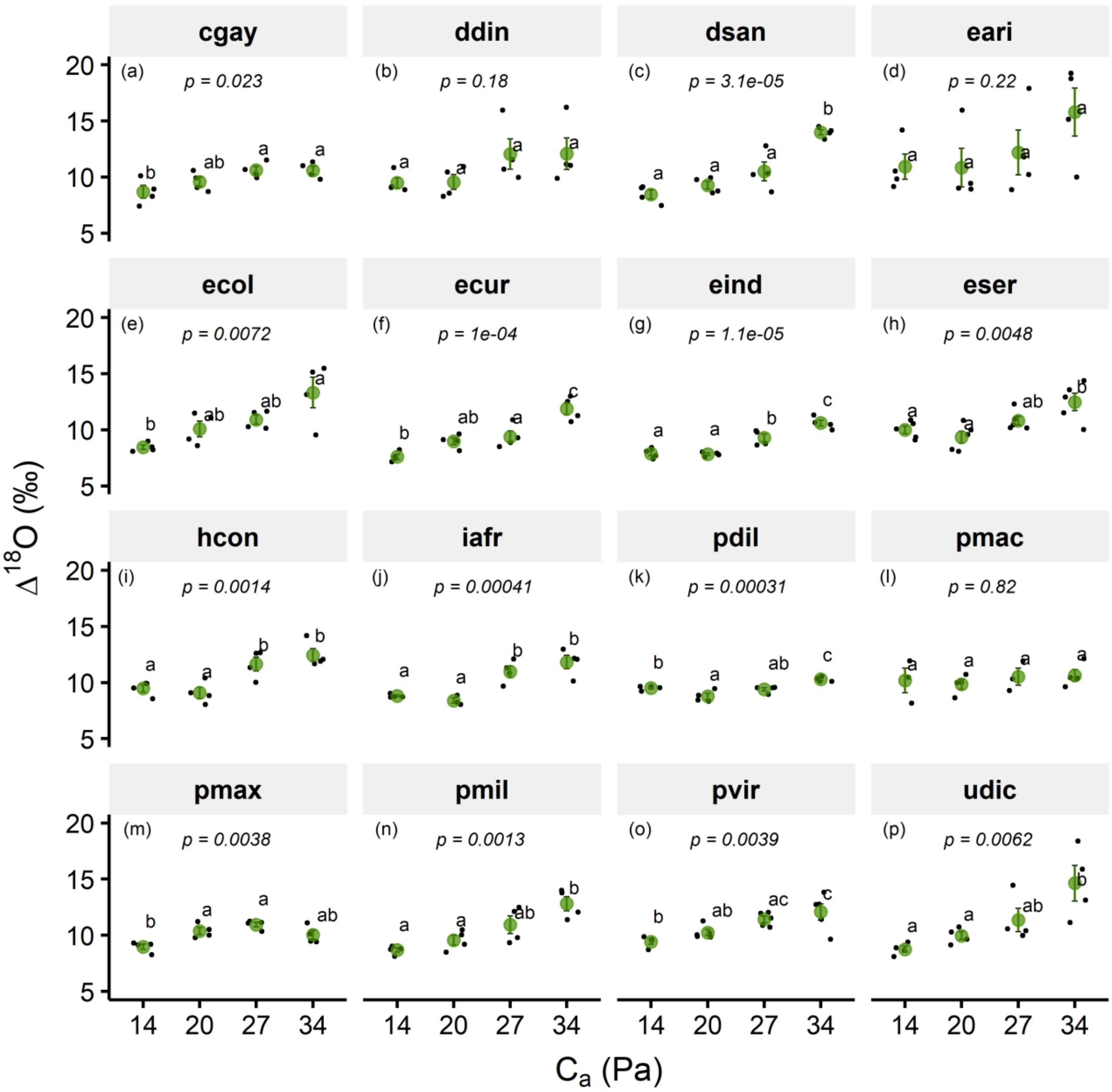

Fig. S11 Response of leaf net $C^{18}O^{16}O$ discrimination ($\Delta^{18}O$) to changes in pCO_2 inside leaf chamber (C_a) in 16 diverse C_4 grasses measured in current study. c. Measurements were performed at constant light (photosynthetic photon flux density (PPFD) = 1200 µmol m^{-2} s^{-1}) and leaf temperature (25°C). Values in each panel represent mean ± 1 SE (green color) with n = 3-6. Black points indicate the replicate values for each species and CO_2 level. Response of $\Delta^{18}O$ to CO_2 S for each species is plotted in separate panel. Species code has been indicated by first letter of genera and first three letters of species (see Table 1 for full names of species). *P*-values from one-way ANOVA along with Tukey's letters are shown. Results of two-way ANOVA are given in Table 2.

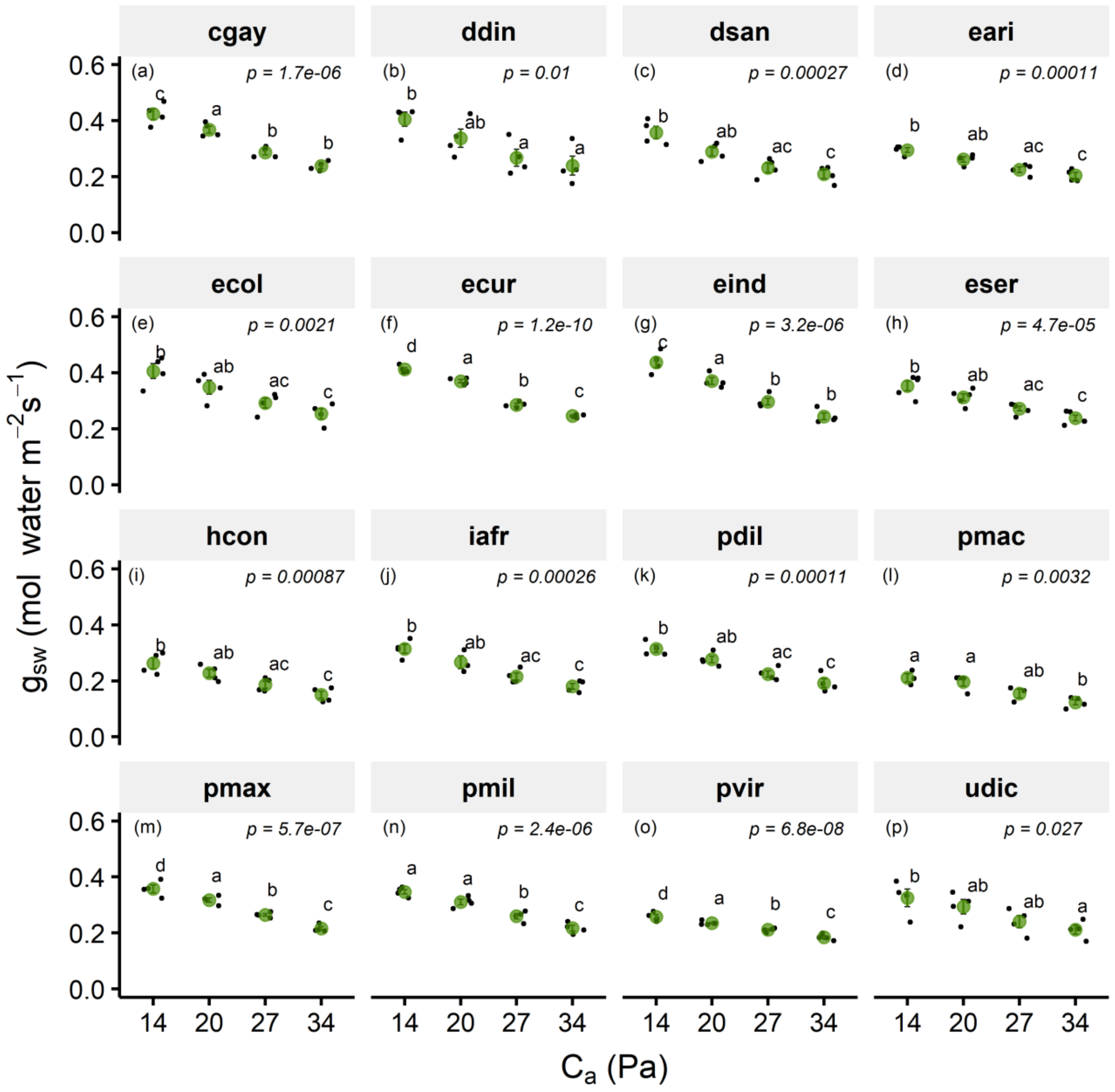

Fig. S12 Response of stomatal conductance to water (g_{sw}) to changes in pCO$_2$ inside leaf chamber (C_a) 16 diverse C_4 grasses measured in current study. Data for each of the species is shown separately from panel a to p. Measurements were performed at constant light (photosynthetic photon flux density (PPFD) = 1200 µmol m^{-2} s-^{1}) and leaf temperature (25°C). Values in each panel represent mean ± 1 SE (green color) with n = 3-6. Black points indicate the replicate values for each species and CO_2 level. Response of g_{sw} to CO_2 S for each species is plotted in separate panel. Species code has been indicated by first letter of genera and first three letters of species (see Table 1 for full names of species). *P*- values from one-way ANOVA along with Tukey's letters are shown. Results of two-way ANOVA are given in Table 2.

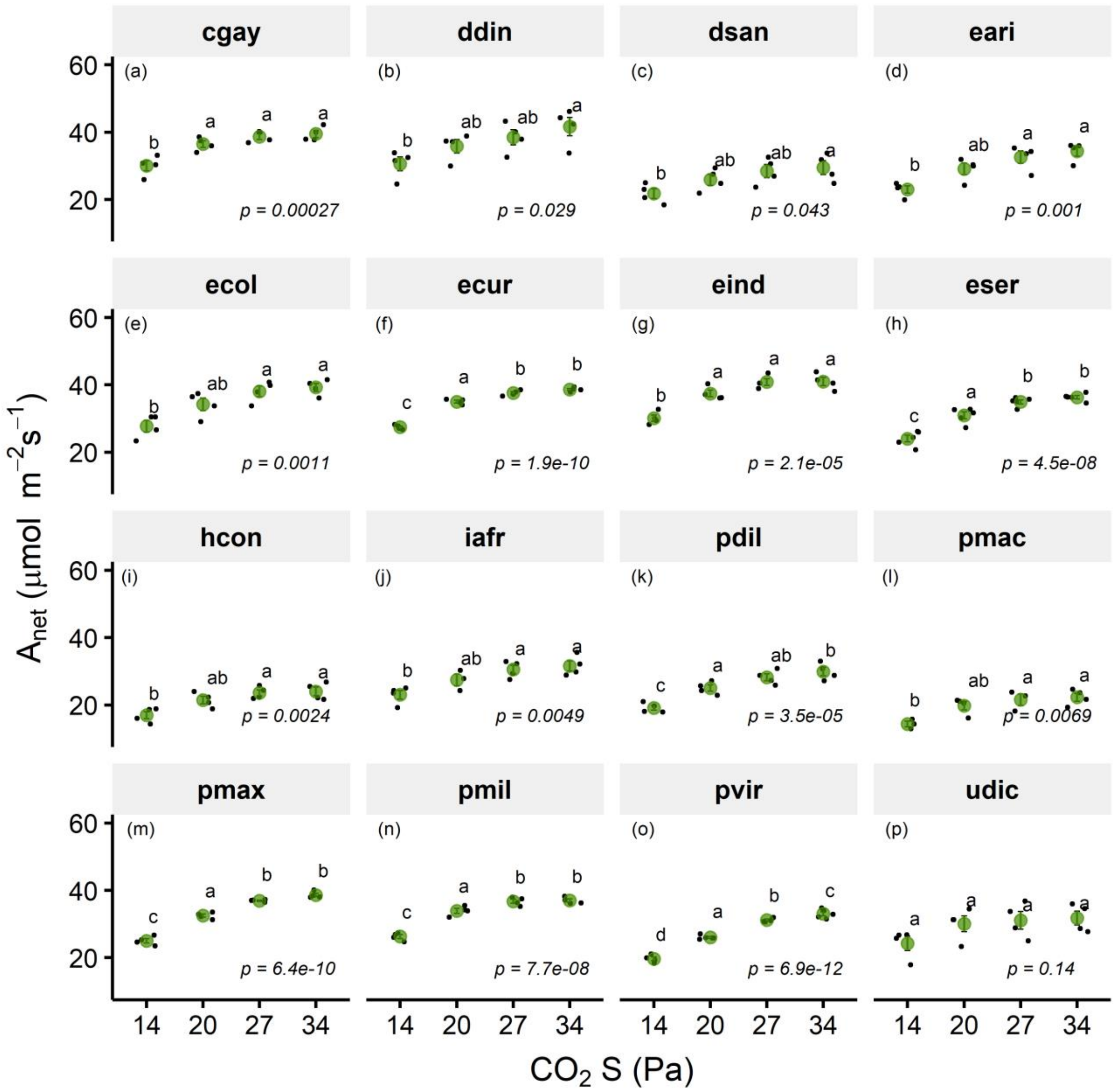

Fig. S13 Response of net CO_2 assimilation rates (A_{net}) to changes in *p*CO_2 inside leaf chamber (C_a) in 16 diverse C_4 grasses measured in current study. Data for each of the species is shown separately from panel a to p. Measurements were performed at constant light (photosynthetic photon flux density (PPFD) = 1200 µmol m^{-2} s^{-1}) and leaf temperature (25°C). Values in each panel represent mean ± 1 SE (green color) with *n* = 3-6. Black points indicate the replicate values for each species and CO_2 level. Response of A_{net} to CO_2 S for each species is plotted in separate panel. Species code has been indicated by first letter of genera and first three letters of species (see Table 1 for full names of species). *P*- values from one-way ANOVA along with Tukey's letters are shown. Results of two-way ANOVA are given in Table 2.

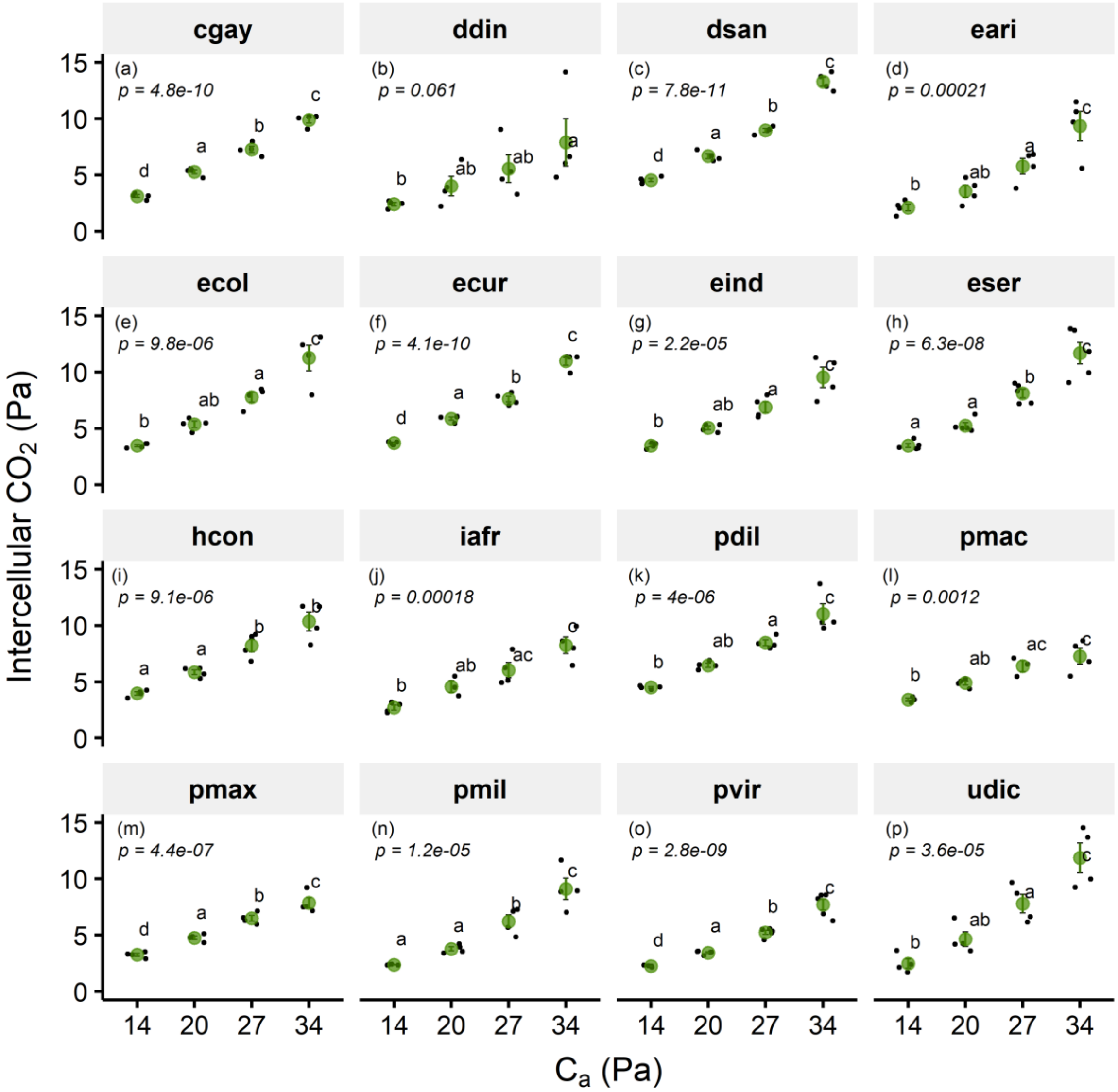

Fig. S14 Response of leaf intercellular CO_2 concentration (C_i) to changes in *p*CO_2 inside leaf chamber (C_a) in 16 diverse C_4 grasses measured in current study. Data for each of the species is shown separately from panel a to p. Measurements were performed at constant light (photosynthetic photon flux density (PPFD) = 1200 µmol m^{-2} s^{-1}) and leaf temperature (25°C). Values in each panel represent mean ± 1 SE (green color) with n = 3-6. Black points indicate the replicate values for each species and CO_2 level. Response of C_i to CO_2 S for each species is plotted in separate panel. Species code has been indicated by first letter of genera and first three letters of species (see Table 1 for full names of species). *P*- values from one-way ANOVA along with Tukey's letters are shown. Results of two-way ANOVA are given in Table 2.

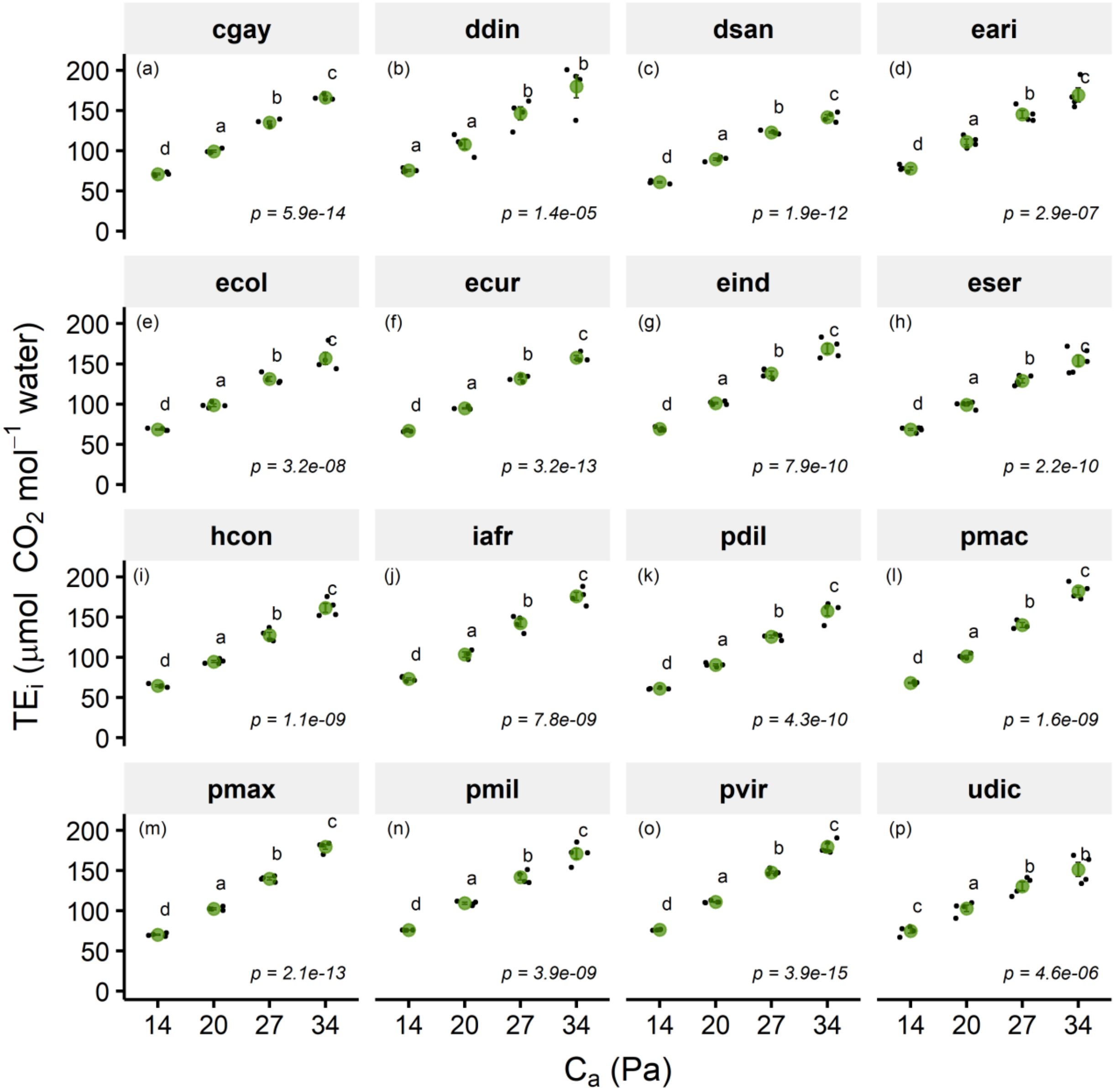

Fig. S15 Response of leaf-level water-use efficiency ($TE_i = A_{net}/g_{sw}$) to changes in pCO_2 inside leaf chamber (C_a) in 16 diverse C_4 grasses measured in current study. Data for each of the species is shown separately from panel a to p. Measurements were performed at constant light (photosynthetic photon flux density (PPFD) = 1200 μmol m^{-2} s^{-1}) and leaf temperature (25°C). Values in each panel represent mean ± 1 SE (green color) with *n* = 3-6. Black points indicate the replicate values for each species and CO_2 level. Response of TE_i to CO_2 S for each species is plotted in separate panel. Species code has been indicated by first letter of genera and first three letters of species (see Table 1 for full names of species). *P*- values from one-way ANOVA along with Tukey's letters are shown. Results of two-way ANOVA are given in Table 2.

References

Boyd RA, Gandin A, Cousins AB. 2015. Temperature Responses of C_4 Photosynthesis: Biochemical Analysis of Rubisco, Phospho*enol*pyruvate Carboxylase, and Carbonic Anhydrase in *Setaria viridis*. *Plant Physiology* **169**(3): 1850-1861.

Evans J, Caemmerer S, Setchell B, Hudson G. 1994. The Relationship Between CO_2 Transfer Conductance and Leaf Anatomy in Transgenic Tobacco With a Reduced Content of Rubisco. *Functional Plant Biology* **21**(4): 475-495.

Kaiser HF. 1960. The application of electronic computers to factor analysis. *Educational and Psychological Measurement* **20**(1): 141-151.

Le S, Josse J, Husson F. 2008. FactoMineR: An R Package for Multivariate Analysis. *Journal of Statistical Software* **25**(1): 1 - 18.

Ogee J, Wingate L, Genty B. 2018. Mesophyll conductance from measurements of $C^{18}OO$ photosynthetic discrimination and carbonic anhydrase activity. *Plant Physiology* **178**: 728-752.

Pathare VS, Koteyeva N, Cousins AB. 2020. Increased adaxial stomatal density is associated with greater mesophyll surface area exposed to intercellular air spaces and mesophyll conductance in diverse C_4 grasses. *New Phytologist* **225**(1): 169-182.

Sharwood RE, Ghannoum O, Kapralov MV, Gunn LH, Whitney SM. 2016. Temperature responses of Rubisco from Paniceae grasses provide opportunities for improving C_3 photosynthesis. *Nature Plants* **2**: 16186.

Ubierna N, Gandin A, Boyd RA, Cousins AB. 2017. Temperature response of mesophyll conductance in three C_4 species calculated with two methods: ^{18}O discrimination and *in vitro* V_{pmax}. *New Phytologist* **214**(1): 66-80.

von Caemmerer S. 2000. *Biochemical Models of Leaf Photosynthesis*. CSIRO Publishing, Collingwood, Australia.
